# A digital application and augmented physician rounds reduce postoperative pain and opioid consumption after primary total knee replacement (TKR): a randomized clinical trial

**DOI:** 10.1186/s12916-022-02638-0

**Published:** 2022-12-05

**Authors:** Julia Stuhlreyer, Christian Roder, Florian Krug, Christian Zöllner, Herta Flor, Regine Klinger

**Affiliations:** 1 Department of Anesthesiology, University Medical Centre Hamburg-Eppendorf, Hamburg, Germany; 2 Department of Orthopedics and Trauma Surgery, Schön Clinic Hamburg Eilbek, Hamburg, Germany; 3 Institute of Cognitive and Clinical Neuroscience, Central Institute of Mental Health, Medical Faculty Mannheim, Heidelberg University, Mannheim, Germany

**Keywords:** Opioid reduction, Pain management, e-Health, Open-hidden medication paradigm, Placebo effect, Postoperative pain, TKR, Blended care

## Abstract

**Background:**

Severe postoperative pain not only is a considerable burden for patients but also leads to overprescription of opioids, resulting in considerable health concerns. The remarkable development of new technologies in the health care system provides novel treatment opportunities in this area and could exploit the additional placebo effect, provide added value for patients, and at the same time support hospital staff. We aimed to test the pain- and opioid intake-reducing effects of enhanced postoperative pain management by boosting pain medication by using a technical application and/or augmented physician rounds.

**Methods:**

In a four-arm, randomized clinical trial, 96 patients (24 patients per group) scheduled for a total knee replacement (TKR) were randomized into four groups for four postoperative days: an “application” group (APP) with information via an iPad-based application; a “doctor” group (DOC) with augmented physician rounds; a combination group (APP+DOC), which received both interventions; and a “treatment as usual” group (TAU) as a baseline with no additional intervention besides the standard care which consists of standardized medication, regular physician rounds, and physiotherapy. Postoperative pain and opioid requirements pre- and postoperatively until hospital discharge were recorded.

**Results:**

The difference between post- and preoperative pain was significantly different between the groups (*P=*.02, partial η^2^=.10). APP+DOC experienced greater postoperative pain relief than DOC (mean: 2.3 vs. 0.7, 95% CI: 0.08–3.09; *P*=.04) and TAU (mean 2.3 vs. 0.1; 95% CI: 0.69–3.71; *P*=.005), respectively, the difference compared to APP (mean 2.3 vs. 1.7; 95% CI −1.98–1.76) was not significant. Opioid consumption differed significantly between groups (*P*=.01, partial η^2^=.12). APP+DOC (72.9 mg) and DOC (75.4 mg) consumed less oxycodone than APP (83.3 mg) and TAU (87.9 mg; 95% CI: 2.9–22.1; *P*=.003). APP+DOC consumed significantly less oxycodone than DOC (*d*=0.2–0.4). There were no significant group differences in NSAID and Morphine sulfate consumption. Patients in APP+DOC were more satisfied with their treatment than patients in TAU (*P*=.03, partial *η*^*2*^*=*.09).

**Conclusions:**

The combination of an innovative digital app, which implements open drug administration and augmented physician rounds that support the doctor–patient relationship can significantly improve postoperative pain management.

**Trial registration:**

The protocol was approved by the local ethics committee of the ethical commission of the German Psychological Society (Deutsche Gesellschaft für Psychologie; DGPs). The study was registered at DRKS.de (identifier: DRKS00009554).

**Supplementary Information:**

The online version contains supplementary material available at 10.1186/s12916-022-02638-0.

## Background

Severe postoperative pain not only is a considerable burden for patients but also leads to overprescription of opioids, resulting in considerable health concerns [1, 2]. Given the current opioid crisis [3] which is characterized by opioid misuse and addiction, the postoperative prescription of opioids needs to be minimized. Hence, new treatment strategies to reduce opioid prescriptions are urgently needed [2, 4]. The remarkable development of new technologies in the health care system provides novel treatment opportunities in this area and could provide added value for patients and at the same time support hospital staff and reduce costs [5, 6].

Opioid overuse usually starts immediately after surgery [7]. However, no adequate and viable alternatives to opioids exist, making it difficult to decrease their use [8]. Currently, there are no clear guidelines or ways to reduce postoperative opioid prescriptions without increasing postoperative pain [9].

Digital devices offer patients the advantage of access to important information related to their health and pain status; this can support the efficacy of their medication intake. Digital medical devices could potentially integrate the concept of “open medication” into the clinical routine [10]. Open medication, in contrast to hidden medication, is characterized by overtly administered analgesics; patients are fully aware of the type and amount of the drug they are taking as well as its effects [11, 12]. Measures that improve the perception of a drug (e.g., sight and smell) and the knowledge about it improve its efficacy. This “additional placebo effect” is inherent in every analgesic treatment [11]. However, it is questionable whether the mere use of technical aids without interpersonal relationships is effective for treatment outcomes. The patient–doctor relationship plays an important role in open medication and consequently the efficacy of pain management [13–15]. Augmented physician rounds, where the physician pays added attention to the patients’ concerns, could also reduce postoperative pain and opioid use and provide an additional placebo effect and thus pain reduction above that inherent in the medication [11, 12]. Therefore, the influence of enhanced physician rounds should be investigated in the context of open medication and postoperative pain to boost the analgesic effect.

Exploiting the additional placebo effect is especially important after surgeries from which patients experience severe postoperative pain. Total knee replacement (TKR) surgery is usually followed by severe pain for 15–30% of patients [16]. To date, inpatient treatment as usual (TAU) for TKR in our institution consists of medical treatment, regular physician rounds, and physiotherapy for approximately 4 days. Together with other drugs, analgesics are usually distributed through a drug dispenser labeled “morning,” “midday,” and “evening.” Patients do not know which of the drugs are the analgesics, and usually, they do not receive specific information about exactly what effects their pain medications have, when the effects occur, and how long they last. Exact medication intake times are not provided; thus, the true medication intake has a high inter- and intraindividual variance.

Our aim was to improve the postoperative pain experience and to reduce opioid consumption in patients with postoperative pain after TKR. Based on findings from the research field of open medication, we investigated whether openly administered medication implemented through a newly developed digital application (app) combined with augmented physician rounds leads to greater pain relief (primary outcome), reduction in oxycodone consumption, and an increase in self-rated treatment satisfaction with postoperative medication (secondary outcomes).

## Methods

### Trial design and oversight

This randomized controlled clinical trial (RCT) included three treatment groups and one therapy as usual control group (see the “Study design and materials” section). These groups comprised 96 patients (24 patients per group) undergoing TKR at a German hospital center in Hamburg.

The study was conducted in accordance with the principles of the Declaration of Helsinki, and the protocol was approved by the local ethics committee of the ethical commission of the German Psychological Society (Deutsche Gesellschaft für Psychologie; DGPs). The study was registered at DRKS.de (identifier: DRKS00009554).

### Participants

Patients 18 years of age or older were eligible to participate in the study if they were undergoing TKR due to knee osteoarthritis. Patients were excluded if they were cognitively impaired, had insufficient command of the German language, had a diagnosed mental disorder (according to the International Classification of Diseases, Tenth Revision (ICD-10 [17])), when they declared that they consumed opioids regularly, any consciousness-impairing substances (e.g., psychoactive drugs, including illegal drugs), or suffered from pain requiring a special causative medical treatment (e.g., cancer-related pain). All participants participated voluntarily; they were informed about the study and provided written informed consent. Furthermore, only one patient per hospital room could participate in the study to exclude potential positive or negative effects by observing a roommate.

### Anesthesia and postoperative standard treatment and care (therapy as usual)

All patients included in the study received the standard operating procedure of anesthesia and postoperative pain management, which included general anesthesia or spinal local anesthesia, physician rounds, postoperative physiotherapy, and postoperative pain medication according to the German Guidelines (AWMF, Version 2016). Anesthesia was standardized and equal between groups. The patients were able to self-determine their pain medication intake within predetermined maximum limits, and the medications included conventional NSAID/COX-2-selective inhibitors, paracetamol, and opioids and morphine sulfate. However, patients had the option to deviate from this regimen on an individual basis and take less medication if pain was less severe. In the case of insufficient pain relief, patients had the option to request more analgesics. In consultation with the physician, NSAIDs and oxycodone intake could then also be increased on an individualized basis (PRN) and was therefore defined as the second outcome. In our study, all groups received the same type of anesthesia (see Table 1); only the interventions to be studied varied among the four groups.

**Table 1:** Demographic baseline characteristics^a,b^

Variable	APP (***N*** = 24)	DOC (***N*** = 24)	APP+DOC (***N*** = 24)	TAU (***N*** = 24)	***p***
***Demographics***					
**Age — year**	65 ± 12	69 ± 9	66 ± 9	71 ± 8	.08
**Female sex — no. (%)**	14 (58)	14 (58)	14 (58)	14 (58)	
***Clinical knee pain***					
Preoperative clinical knee pain without analgesics **—** NRS	6.3 ± 1.6	7.0 ± 1.9	6.6 ± 1.9	6.3 ± 2.7	.63
**Missing — no. (%)**	1 (4)	0 (0)	0 (0)	1 (4)	
*Acute knee pain*					
Preoperative acute knee pain one day prior to surgery **—** NRS	4.3 ± 2.4	3.5 ± 2.6	4.5 ± 2.5	3.16 ± 2.6	.25
**Missing — no. (%)**	4 (17)	2 (8)	3 (13)	5 (21)	
***Expectation about postoperative pain***					
Expected knee pain with analgesics **—** NRS	4.0 ± 2.2	2.9 ± 2.3	4.5 ± 2.8	3.0 ± 2.2	.20
Expected wound pain with analgesics **—** NRS	3.5 ± 2.6	3.2 ± 3.0	2.4 ± 2.5	2.5 ± 2.4	.39
**Missing — no. (%)**	1 (4)	0 (0)	0 (0)	0 (0)	
***Self-rated functional capacity***					
Subjective functional capacity without analgesics **—** NRS	6.1 ± 2.1	7.0 ± 2.8	6.1 ± 3.0	6.3 ± 2.2	.58
**Missing — no. (%)**	1 (4)	0 (0)	0 (0)	1 (4)	
***Self-rated physical functional capacity: expectations***					
Expected functional capacity with analgesics **—** NRS	4.9 ± 3.0	4.4 ± 3.2	4.6 ± 2.7	4.8 ± 2.6	.96
**Missing — no. (%)**	1 (4)	1 (4)	1 (4)	0 (0)	
***Questionnaires***					
Lequesne Index (questions related to pain, ability to walk, and coping with everyday life)	11.8 ± 3.7	12.3 ± 4.5	13.4 ± 3.7	13.1 ± 3.8	.52
**Missing — no. (%)**	0 (0)	2 (8)	2 (8)	3 (13)	
*PHQ***—***4* (health questionnaire for patients consisting of the PHQ-2 and GAD-2)					
PHQ-2 **—** no. (%) (questions related to depression)					.68
**< 3**	17 (71)	18 (75)	16 (67)	16 (67)	
**≥ 3**	5 (21)	3 (13)	5 (21)	2 (8)	
**Missing — no. (%)**	2 (8)	3 (13)	3 (13)	6 (25)	
GAD-2 **—** no. (%) (questions related to generalized anxiety)					.85
**< 3**	21 (88)	19 (79)	17 (71)	18 (75)	
**≥ 3**	1 (4)	2 (8)	3 (13)	2 (8)	
**Missing — no. (%)**	2 (8)	3 (13)	4 (17)	4 (17)	
*FSS* (questions related to pain-related self-instructions)					
**Catastrophizing self-instructions**	1.4 ± 1.0	1.3 ± 1.2	1.4 ± 1.1	1.3 ± 1.1	.96
**Missing — no. (%)**	2 (8)	3 (13)	3 (13)	4 (17)	
**Active coping self-instructions**	2.6 ± 1.3	2.4 ± 1.3	2.4 ± 1.3	2.7 ± 1.2	.83
**Missing — no. (%)**	2 (8)	2 (8)	2 (8)	3 (13)	
Stanford Expectations of Treatment Scale (*SETS*)					
**SETS positive expectations**	2.2 ± 0.8	2.6 ± 0.9	2.1 ± 0.7	2.4 ± 1.1	.38
**Missing — no. (%)**	0 (0)	2 (8)	3 (13)	4 (17)	
**SETS negative expectations**	5.0 ± 1.3	4.9 ± 1.4	4.9 ± 1.4	4.5 ± 1.5	.69
**Missing — no. (%)**	1 (4)	2 (8)	3 (13)	4 (17)	
**Medication scheme**					
Standard	20	20	19	23	
Metamizole intolerance	1	1	2	0	
Renal insufficiency	3	3	3	1	
**Anesthesia**					
General anesthetic (%)	9 (38)	9 (38)	6 (25)	2 (8)	
Spinal anesthesia (%)	15 (63)	13 (54)	18 (75)	20 (83)	
Missing **—** no. (%)	0 (0)	2 (8)	0 (0)	2 (8)	

^a^ Baseline characteristics are related to preoperative pain (NRS), postoperative pain expectations (NRS), functional capacity, self-reported disabilities (Lequesne Index), questions related to depression (PHQ-2), generalized anxiety (GAD-2), pain-related self-instructions (FSS), and treatment expectations (SETS)

^b^ Baseline data are based on the full population indicated in the column headings. If data regarding the baseline characteristics was not available for all patients, the missing numbers for these characteristics are presented in the lines labeled “missing.” The characteristics of the patients at baseline were well balanced between all trial groups. Percentages may not total 100 because of rounding. Plus-minus values are standard deviations (*SD*s*)*

### Study design and materials

**Fig. 1:**
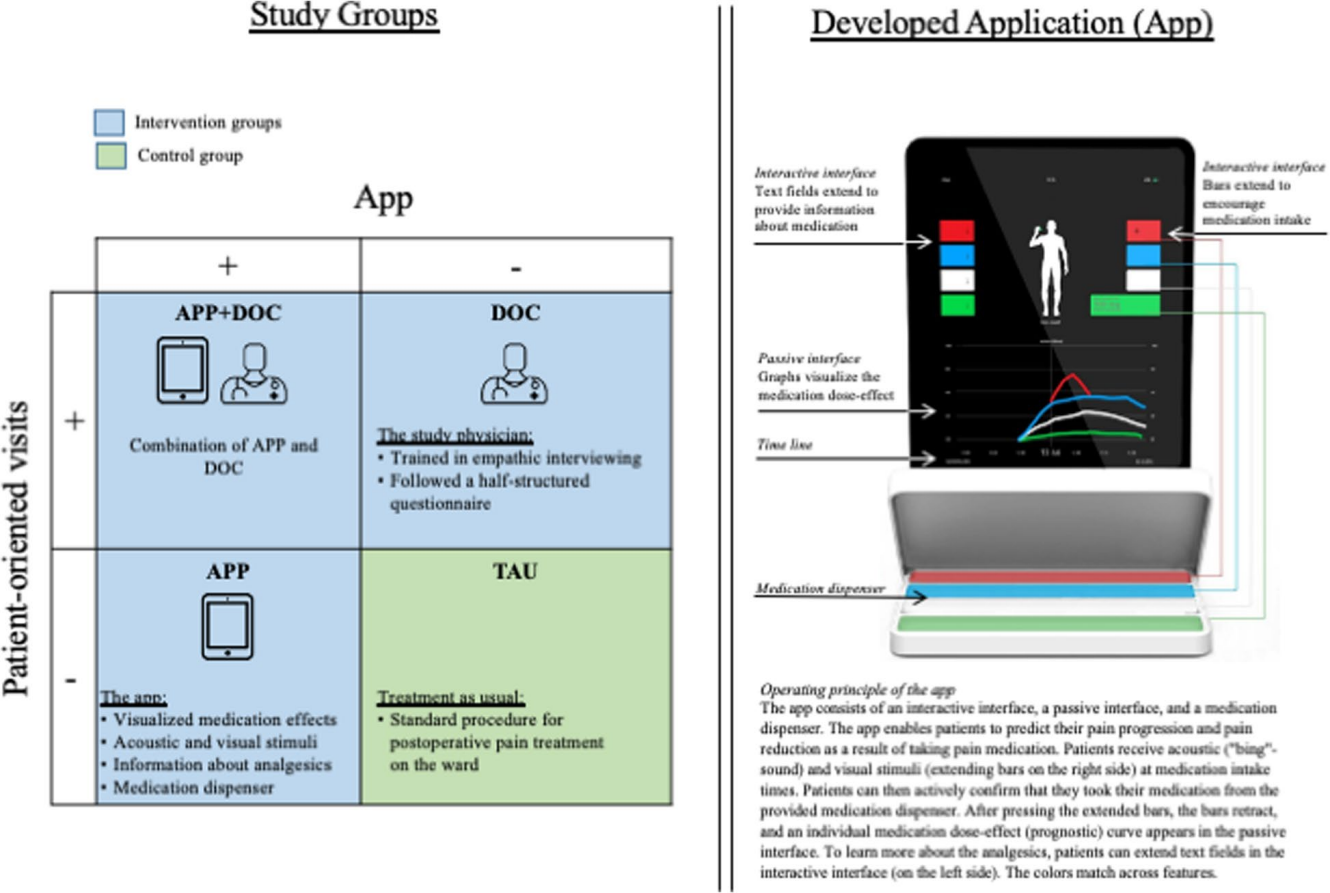
Study groups (left side) and the application that was developed (right side)

To investigate the additive and synergistic effects of digital health and augmented physician rounds, we implemented a 2×2 full factorial study design (Fig. 1). This study design is thus far the only possibility to examine the effects of two new interventions (APP and DOC) separately and in their interaction (APP+DOC) in comparison to the standard procedure (TAU). Moreover, we investigated another group to control for a potential bias effect deriving from the circumstance that the app reminded the patients to take their medication at exact time points. In this group, the patients also received their medications on a time-contingent basis but without APP and DOC. Since this group did not differ from the TAU group and had no influence on the results, we did not include it in the following presentation of the results in favor of a more concise presentation of the study. However, the results can be found in the appendix (see Doc S1).

The treatment phase lasted for four postoperative days, beginning the first day after surgery. Patients received their postoperative pain medication openly in the APP, DOC, and APP+DOC treatment groups.

Open medication was implemented either through augmented physician rounds and/or a specific app developed for the study (Fig. 1). The treatment groups (in contrast to the control group) were provided with detailed information through the app, the physician in a personal atmosphere, or both. This information enabled patients to understand the effects of their pain medication in detail and to anticipate the course of action over the next few days.

#### APP: Open medication via the iPad app

The patients in the first treatment group (APP) received written and visualized information about their pain medication via the newly developed digital app (Fig. 1). The app enabled patients to predict their pain progression and their pain reduction as a result of taking pain medication. The app asked that the patients confirm intake of the medication and generate a prognostic curve of its analgesic effects based on its pharmacokinetics. The prognostic curve was visualized on an iPad screen (Fig. 1), and this forecast of the analgesic effects of the drugs allowed the patient to develop certainty about the course of their pain in the next hours. In addition, it reminded patients of specific times to take their medications.

#### DOC: Open medication via augmented physician rounds

In the second treatment group (DOC), the same information, but without any visualization, was conveyed via the physician for five minutes daily. He was a staff member of the clinic where the TKR was performed and the study physician of our research team. During his enhanced physician rounds with the patients in the DOC, he responded emphatically and individually to the patients and encouraged them to ask any questions, including questions about their surgery and pain medications.

#### APP+DOC: Open medication via the iPad app and augmented physician rounds

In the third treatment group (APP+DOC), information was conveyed via the app and the responsible physician (augmented physician rounds). In this group setting, the physician provided information about pain medication tailored to individuals and visualized its pain-reducing effects by using the prognostic curve of the iPad app. Moreover, he responded emphatically to patients’ annotations and encouraged them to ask further questions.

#### TAU: Therapy as usual

In contrast, patients in the control group received medication through standard hospital procedures; this involved the use of a medicine dispenser of the standard hospital medicine dispenser labeled “morning,” “midday,” and “evening.” The patients received standard physician rounds without tailored information. TAU was used to provide a baseline against which the additional benefit of the investigated intervention could be determined.

#### Course of the study

The course of the study can be comprehended in more detail in the study protocol (Doc S2). Eligible patients were informed about the study one day prior to surgery. After they had signed the informed consent, the first interview was conducted. This interview collected demographic information, pain ratings, physical function capacity, and the patient’s expectations regarding postoperative acute knee pain. Next, the 96 patients were randomly assigned to one of the four groups (24 patients per group) and received a preoperative questionnaire that assessed preoperative pain, expectations about postoperative pain, physical functional capacity and status, self-reported disabilities, and questions with respect to depression, generalized anxiety, pain-related self-instructions (catastrophizing), and treatment expectations (Table 1). After the baseline assessment, patients were assigned to their treatment group and informed about it. After the surgery, patients received their treatment according to their group allocation. Based on medical guidelines, all patients received metamizole (dipyrone), celecoxib, oxycodone, and morphine (rescue medication) as postoperative analgesics. The only deviations in standard medication were for known preexisting conditions (Table 2). All patients received their medication according to the scheme, and potential deviations did not differ between the groups (see Table 1). Therefore, the treatment regimen did not affect the results or the conclusion. During the inpatient stay, all patients completed a pain diary in which they indicated their pain ratings for four consecutive days, beginning the first day after surgery. Finally, a concluding interview assessed postoperative pain ratings and functional capacity. Furthermore, all patients reported their satisfaction about the pain treatment that had been provided.

**Table 2:** Standardized medication plan for postoperative analgesics

Medication plan	Medication	Frequency of intake (per day)	Active ingredient quantity (mg)	Total quantity per day (mg)	Total quantity for 4 days (mg)
Standard	Metamizole (Dipyrone)	4	500	2000	8000
	Celecoxib	2	200	400	1600
	Oxycodone	2	10	20	80
	Morphine (rescue medication)	Max. 6	10	Max. 60	Max. 240
In case of metamizole (dipyrone) intolerance	Paracetamol	8	500	4000	16,000
	Celecoxib	2	200	400	1600
	Oxycodone	2	10	20	80
	Morphine (rescue medication)	Max. 6	10	Max. 60	Max. 240
In case of cardiac insufficiency	Metamizole (Dipyrone)	4	500	2000	8000
	Ibuprofen	3	600	1800	7200
	Oxycodone	2	10	20	80
	Morphine (rescue medication)	Max. 6	10	Max. 60	Max. 240
In case of renal insufficiency	Metamizole (Dipyrone)	4	500	2000	8000
	Oxycodone	2	10	20	80
	Morphine (rescue medication)	Max. 6	10	Max. 60	Max. 240

### Trial procedures

All patients were randomly assigned to one of the four groups. Group assignment was performed by an independent researcher who was not related to the study and who randomly drew pieces of paper from four piles that were stratified by age and sex ((1) female and ≤ 70 years of age; (2) male and ≤ 70 years of age; (3) female and > 70 years of age; and (4) male and > 70 years of age). To eliminate unintended bias, all the pieces of paper were folded and were identical from the outside. The group allocation was communicated to the researcher after the baseline assessment. Regardless of their group allocation, all patients received the usual care, which included hospitalization for 4 days after the surgery, analgesics according to medical guidelines, standard physician rounds, and physiotherapy. The healthcare staff (nurses, physiotherapists, physicians) of the clinical routine care were not informed and therefore not aware of the group allocation. Patients in the APP groups (APP, APP+DOC) were asked at the fourth day after the surgery if they made use of the information system.

### Outcomes

We tested the interventions with patient-related outcome measures. The primary outcome was self-documented average postoperative knee pain for 4 days following the TKR rated on a numerical rating scale (NRS; 0 = no pain; 10 = worst pain imaginable). Hence, the course of pain based on the pain ratings (morning, midday, and evening) for four consecutive postoperative days was analyzed. Additionally, pain relief was assessed by calculating the difference between the pain level one day prior to the surgery (directly after admission) and the pain level experienced on the fourth postoperative day. This outcome measure was chosen because preoperative pain influences postoperative pain and the desire for pain relief. Hence, postoperative pain is not independent of preoperative factors and should be analyzed in accordance with that context. Baseline pain values should be assessed to have an indicator against which potential treatment effects can be assessed [18].

Secondary outcomes were postoperative oxycodone consumption and self-reported treatment success regarding expectation fulfillment. The daily course of oxycodone prescription (in mg) was evaluated to assess oxycodone consumption. Additionally, NSAID and morphine sulfate consumption was analyzed. Treatment satisfaction was analyzed through self-reported treatment expectations before the surgery and with the Stanford Expectations of Treatment Scale (SETS) and questions about treatment satisfaction after the surgery [19].

### Statistical analysis

The statistical analysis can be reproduced in more detail in the statistical analysis plan (Doc S3). A power analysis, calculated with G*Power [20], yielded a sample size of 76 to 112 patients for an expected small to medium effect size of 0.4–0.5, two measurement time points, four groups, a level of significance of α=0.05, and a power of 0.95. Thus, we included 24 patients per group for a total of 96 patients. Data are reported as the means with 95% confidence intervals (CIs) unless otherwise specified. We performed a univariate analysis of variance (ANOVA) to determine differences in pain reduction on a NRS after surgery. For all other analyses, we used a repeated measures analysis of variance (rANOVA) to determine changes on a NRS or consumption in mg over time. The Greenhouse–Geisser or Huynh–Feldt correction for the *F* test was used to adjust the degrees of freedom for deviations from sphericity. Hence, the results revealed the interactive effect of time (postoperative days) and group. For significant main and interaction effects, exploratory post hoc analyses for significant results were derived from Fisher’s least significant difference (LSD) tests. Hence, we were interested in comparing all studied groups with each other. For the primary outcome, the multiple imputation method for missing observations was conducted after assessing whether the data were missing at random. For all performed analyses, two-sided *P* values of *P<*.05 were considered statistically significant. To account for multiple comparisons, Bonferroni corrections were applied. Analyses were performed with IBM SPSS Statistics software, version 27.0 (IBM Corp., Armonk, NY, USA).

## Results

### Characteristics of the participants

**Fig. 2:**
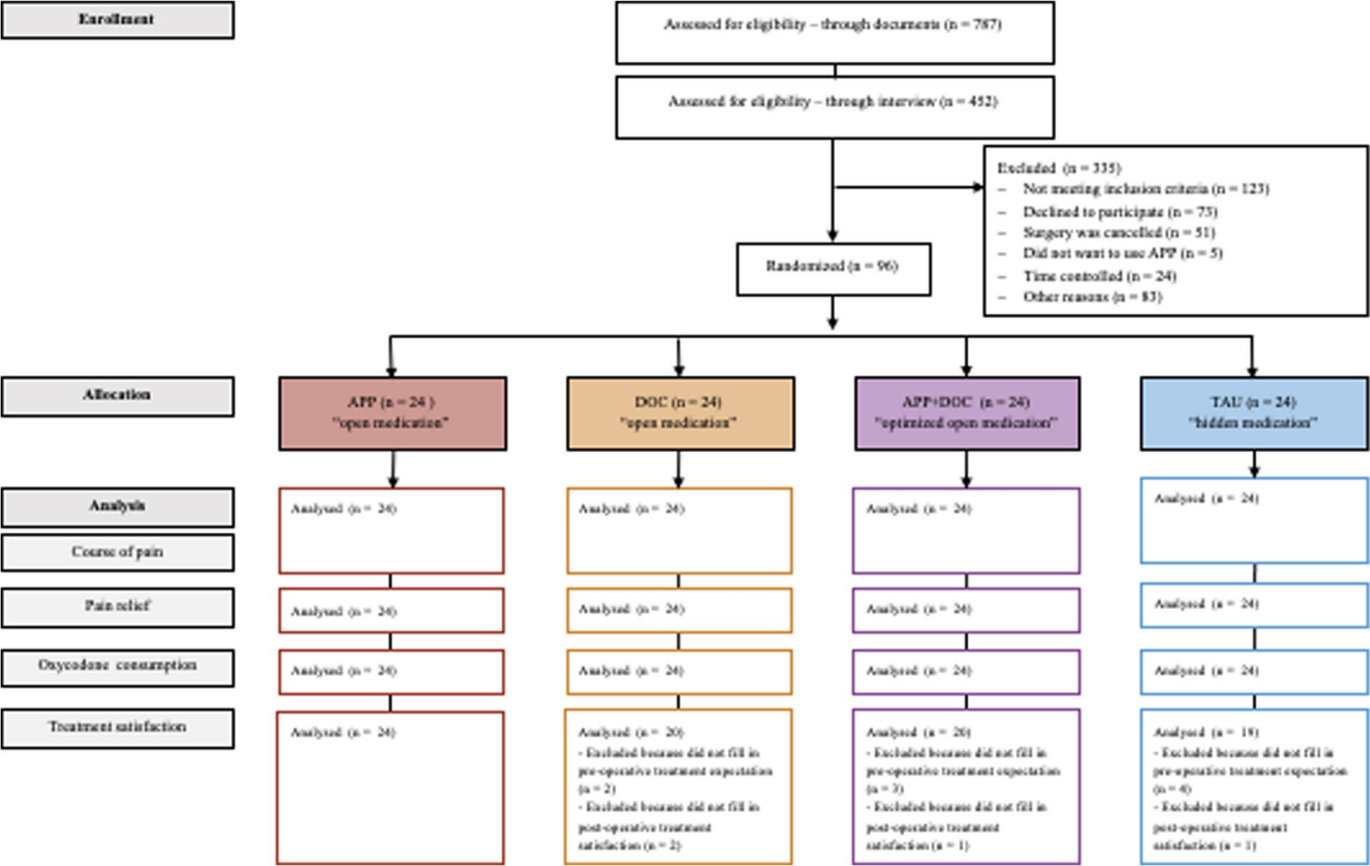
Flowchart of patients in a study of the effects of open medication in patients receiving a total knee replacement

From October 2015 to March 2019, a total of 96 patients (*n*=24 patients per group) at the Centre for Orthopedics and Trauma Surgery in Schoen Clinic Hamburg Eilbek were randomly enrolled into four groups (see CONSORT flowchart; Fig. 2 and Doc S4). Overall, the four groups were balanced with respect to their baseline characteristics (Table 1).

### Primary outcome

**Fig. 3:**
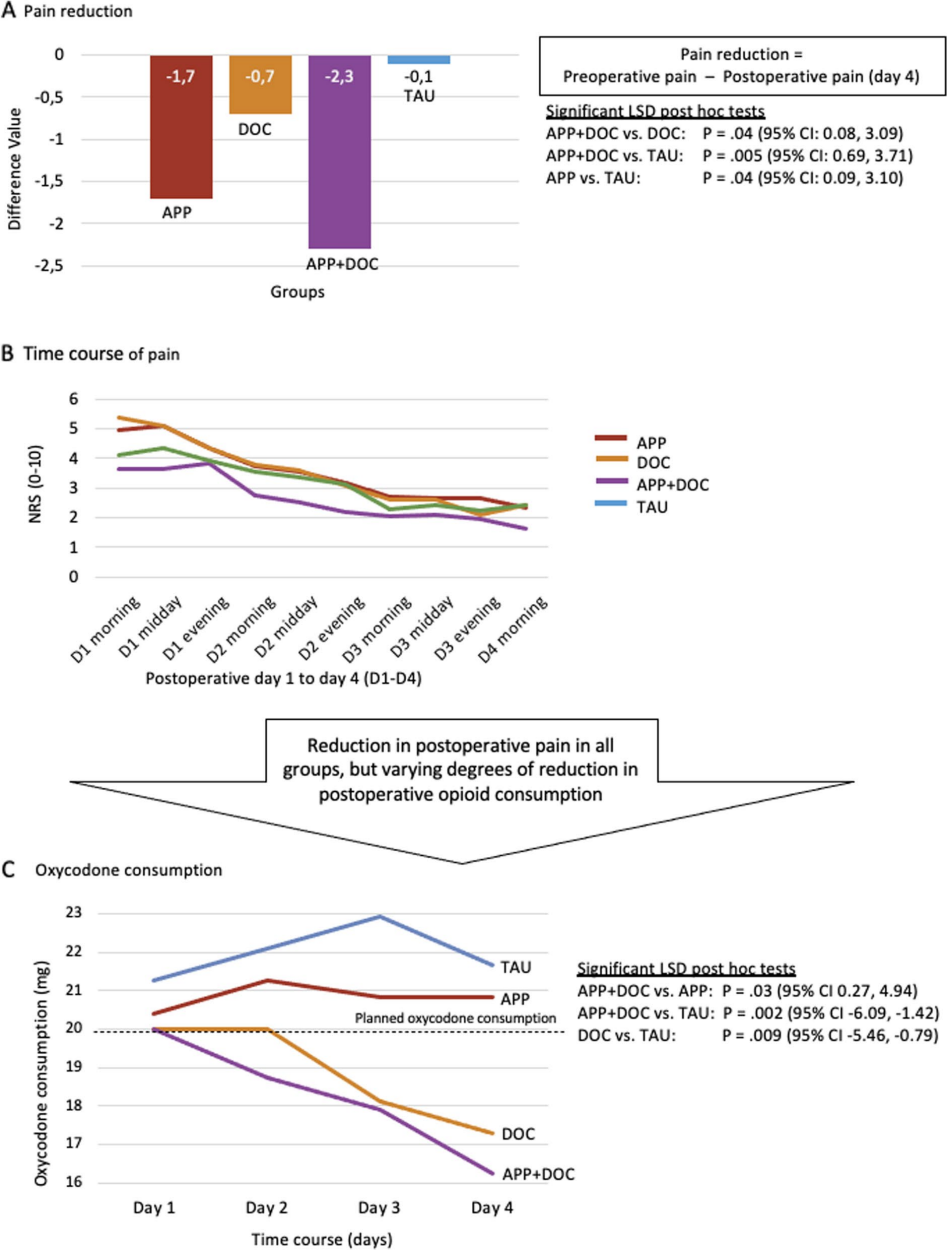
Pain reduction (**A**), time course of pain (**B**), and oxycodone consumption (**C**) in a study of the effect of open medication in patients receiving a total knee replacement

The primary outcome comprises the course of pain over the four consecutive postoperative days. All 96 patients completed the pain ratings in the pain diary. All patients experienced less pain over time within the first four postoperative days (*P*<.001, partial *η*^*2*^ *=*.35; Fig. 3B). However, the course of pain did not significantly differ between groups (*P*=.87, partial *η*^*2*^*=*.02). The relief of postoperative knee pain assessed on an NRS (0–10) significantly differed between groups (*P*=.02, partial η^2^=.10). Specifically, the patients in APP+DOC had a significantly greater reduction in pain than the patients in DOC (*P*=.04) and TAU (*P*=.005; 95% CI: 0.08–3.09; Fig. 3A). APP+DOC experienced 2.3 points less pain (on the NRS) than their preoperative pain ratings, while APP experienced 1.7 points less pain, DOC reported 0.7 points less pain, and TAU experienced 0.1 points less pain than their preoperative pain ratings (Fig. 3A).

### Secondary outcomes

The course of oxycodone consumption significantly differed between the groups in the four postoperative days (*P=*.03, partial η^2^=.04; Fig. 3C). The groups did not significantly differ in oxycodone consumption on the first day (*P*=.26, partial η^2^=.04). However, on the second day (*P*=.048, partial η^2^=.08), third (*P*=.007, partial η^2^=.12) and fourth day (*P*=.02, partial η^2^=.11), the groups with physician support (DOC and APP+DOC) consumed significantly less oxycodone than the groups without physician support (APP and TAU) (Fig. 3C). In addition, APP+DOC consumed less than DOC on the second postoperative day (difference between APP+DOC and DOC: Cohen’s *d*=0.4).

The medication schedule provided up to a maximum of 20 mg of oxycodone daily (in 4 days, a total maximum of 80 mg of oxycodone, Table 1). The average quantity of oxycodone consumption significantly differed between groups (*P*=.01, partial η^2^=.12; Fig. 3C). Specifically, TAU consumed significantly more oxycodone than DOC (*P*=.01; 95% CI: 0.79–5.46) and APP+DOC (*P*=.002; 95% CI: 1.42–6.09). Moreover, APP+DOC showed lower total oxycodone consumption than DOC, with an effect size of Cohen’s d=0.2.

A trend a significant interaction between group and time was shown in the ratings for treatment expectation (*P*=.065, partial *η*^*2*^*=*.09). The post hoc tests revealed a significant difference between APP+DOC and TAU (*P*=.03), with APP+DOC patients indicating higher satisfaction with their treatment than TAU. Novalgin (*F*(6, 185)=0.95, *P*=.46), Celebrex (*F*(3,92)=1, *P*=.40), and morphine sulfate (*F*(2.4, 184)=0.58, *P*=0.77) did not differ significantly between groups.

Additionally, the equal number of patients in the APP and APP+DOC groups made use of the information system in the APP. No patients declared the information system in the APP as negative. Furthermore, a similar number of patients declared the information system as positive.

## Discussion

Our results reveal that the newly developed digital health app, which facilitates open medication, significantly supported postoperative patient-oriented physician rounds. However, the combination of physician rounds with the use of the app led to considerably stronger postoperative pain relief and significantly less oxycodone consumption. Moreover, the app assisted the patient-oriented physician rounds, resulting in higher treatment satisfaction compared to treatment as usual alone. The app and patient-oriented physician rounds led to greater benefits when used in combination than when used alone. For clinical application, it is therefore advantageous to supplement this technical resource with personal contact with the physician.

Digitization and technology are increasingly dominating our healthcare system, and e-health interventions are becoming more and more important in medical treatment [21, 22]. However, there are also concerns that the use of digital apps could come at the expense of interpersonal relationships. As our data demonstrate, digitized and technical tools can have a positive influence on pain treatment outcomes when they provide patients with important information about their prognostic course of pain. One explanation could be that e-health applications enable patients to be more involved and take a more active part in their own treatment [23]. Thus, e-health interventions are also increasingly applied in the perioperative context to prepare patients for surgery [24–26]. Most of these digital applications are developed to deliver only educational or generic information to patients. Additionally, digital tools exist to assist practitioners [27, 28]. To date, no digital application that supports patients in the perioperative pain setting has been developed and scientifically validated or investigated in the context of the patient–physician relationship. According to our data, pain medication showed significantly improved effectiveness when the interaction between the app and augmented physician rounds was exploited.

Postoperative pain decreased for all patients but did not significantly differ between groups. However, postoperative pain was compared to preoperative pain, APP+DOC experienced significantly greater pain relief than DOC and TAU. The difference between pre and postoperative pain in TAU was very small. Other studies often analyze the difference of preoperative pain and pain a couple months after the surgery and can therefore hardly be compared to our results [29, 30]. However, the higher reductions of pain in APP+DOC and APP correspond to our hypotheses. Moreover, the course of oxycodone consumption significantly differed between groups beginning on the third day after surgery. In particular, APP+DOC and DOC consumed approximately 20% less oxycodone than the other two groups. Additionally, satisfaction with treatment expectations and perceived treatment success differed between APP+DOC and TAU. These outcomes support the idea that digital and personal interactions work in a synergistic manner [10, 31].

There are several explanations for the outcomes of the study. By implementing different strategies to administer analgesics, we may have enhanced positive treatment expectations. This is important because treatment expectations strongly influence treatment outcomes [11, 32–37]. Previous research has demonstrated that the placebo effect of analgesics is up to 50% [38]. To build upon positive treatment expectations, we involved the patients in their perioperative treatment. The patients received information from their physician and the app; they were provided written and visual information about their medication via the app and were asked to confirm their medication intake. Confirmation of medication intake facilitated the prognostic curve and the visualization of the drug’s effects. The patients in DOC received the same information (without the app visualization) through their physician. Another explanation for the study outcomes may be the reduction in uncertainty [39]. The app may have conveyed a sense of certainty by providing information about medication intake times, information about analgesics, and a predictive curve to visualize the effects of the analgesics. Uncertainty may also have been reduced in the groups that were supported by patient-oriented doctor rounds. During these augmented physician rounds, the patients were asked to express their concerns and ask questions. The physician built a trust-based relationship with the patients and clarified any uncertainties. Additionally, the physician could modify expectations and directly respond to concerns (e.g., by changing the dose of analgesics). The results are consistent with other studies showing that additional face-to-face physician contact leads to more positive outcomes [40].

The patients in this study were on average 68 years old. Some patients who would have otherwise been eligible could not be included in the study due to age-related cognitive impairment. However, the included patients represent a typical sample of patients who undergo TKR. Despite the advanced age of the patients, utilization of the app was not problematic.

Digital tools developed to help manage acute postoperative pain are numerous [41], although empirical evaluations of their effects are lacking. We showed that digital tools are feasible and effective to improve the treatment of postoperative pain. Furthermore, we demonstrated that open medication, which is a well-investigated approach in the exploitation of placebo effects [42, 43] that are inherent in every analgesic medication [44–49], was superior in an RCT involving patients with postoperative pain. Previous RCTs on open medication [34, 50, 51] investigated patient–clinician communication as a contextual factor and its additional impact on analgesic outcomes [38, 52–55]. To date, there has not been an RCT that has investigated the technical context of open medication. Hence, our approach reveals that open medication can be implemented via technical means to improve analgesic outcomes in experimental trials and clinical treatments.

Some limitations of the current study should be noted. First, oxycodone consumption was assessed only during hospitalization. Hence, no statement about the further course of oxycodone prescription and consumption can be made. A further limiting factor is that patient expectations were assessed only before they were assigned to their group. Therefore, it cannot be evaluated whether the knowledge of group allocation alone influenced treatment expectations. The interaction effect between treatment expectation, treatment satisfaction, and group assignment showed a trend towards with the post hoc tests revealing a significant difference between APP+DOC and TAU. This indicated that the groups differed in treatment satisfaction. The results on treatment satisfaction need to be substantiated in a study with a larger sample size. We assessed postoperative pain in average and did not differentiate between “pain at rest” and “pain during activity.” Due to the reason that patients remarked that their pain differed at rest and during activity, further studies should distinguish between “pain at rest” and “pain during activity.”

Our trial contributes to the current research on methods to reduce postoperative oxycodone consumption without increasing postoperative pain. Given the present opioid crisis, which is based on opioid misuse and addiction [2, 4], the prescription of oxycodone should be minimized without risking an increase in pain. This would not only be beneficial for patients but could also save medical and economic resources [56, 57]. At this time, the opioid crisis can be traced to the postoperative overprescription of opioids [7]. Therefore, a reduction in oxycodone intake in the first days after surgery would be of great benefit to patients and help to reduce the opioid crisis. However, the elimination of opioid use cannot easily be realized due to the lack of alternatives. There are no clear guidelines or methods for reducing postoperative opioid prescriptions without risking increased postoperative pain [9]. The results of this study could support an innovative approach to optimal postoperative pain management.

## Conclusions

In conclusion, this study reveals that patient-centered digital tools can support postoperative pain management and enhance postoperative outcomes, but they cannot replace the effects of augmented physician rounds. The best results were achieved by combining the use of a digital tool (specifically, the app developed for this study) that enhanced open medication and influenced medical treatment expectations with insightful care from the physician to reinforce treatment expectations, establish a relationship based on trust, and clarify uncertainties. The face-to-face, personal contact contributed to an interactive improvement in treatment outcomes and, by combining the strength of the digital tool and patient-oriented doctor contact, improved treatment.

## Supplementary Information

**Additional file 1: Doc S1.** Outcome with Five Groups.

**Additional file 2: Doc S2.** Study Protocol (References: [58–70]).

**Additional file 3: Doc S3.** Statistical Analysis Plan (References: [58–70]).

**Additional file 4: Doc S4.** CONSORT Checklist.

## Data Availability

The datasets used and analyzed during the current study are available from the corresponding author on reasonable request.

## Abbreviations

app: Application
APP+DOC: Application and doctor group
APP: Application group
DOC: Doctor group
LSD: Fisher’s least significant difference test
DGP: German psychological society
ICD-10: International Classification of Diseases, Tenth Revision
NRS: Numeric rating scale
RCT: Randomized controlled clinical trial
rANOVA: Repeated measures analysis of variance
SETS: Stanford Expectations of Treatment Scale
TKR: Total knee replacement
TAU: Treatment as usual
