# Supplementary material for: A digital application and augmented physician rounds reduce postoperative pain and opioid consumption after primary total knee replacement (TKR): a randomized clinical trial
Source: BMC Med. 2022 Dec 5;20:469. doi: 10.1186/s12916-022-02638-0 (PMC9721029; doi:10.1186/s12916-022-02638-0)
Supplement: Supplementary file 1 — Additional file 1: Doc S1. Outcome with Five Groups. [file 12916_2022_2638_MOESM1_ESM.docx]

## Additional file S1

## Document S1 – Outcome with Five Groups

## *3.2 Primary Outcome with Five Groups*

The primary outcome comprises the course of pain for the four consecutive postoperative days. Among the 120 patients, 119 patients (99%) completed pain ratings in the pain diary. All patients experienced less pain over time within the first four postoperative days (*P*<.001, partial *η² =*.37; Fig 3B). However, the course of pain did not differ significantly between groups (*P*=.44, partial *η²=*.03). Among all patients, the relief of postoperative knee pain on an NRS (0–10) differed significantly between groups (*P*=.04, partial η²=.08). Specifically, patients in APP+DOC had a significantly greater reduction in pain than patients in TIME (*P*=.04), DOC (*P*=.05), and TAU (*P*=.006; Fig 3A). The APP+DOC group experienced 2.3 NRS points less pain compared to their preoperative pain ratings, while APP experienced 1.7 NRS points less pain, DOC reported 0.7 NRS points less pain, TIME reported 0.6 NRS points less pain, and TAU experienced 0.1 NRS points less pain than their preoperative pain ratings (Fig 3A).

*3.3 Secondary Outcome*

Among all 120 patients, the course of oxycodone consumption differed significantly between the groups in the four postoperative days (*P=*.04, partial η²=.07; Fig 3C). The groups did not significantly differ in oxycodone consumption on the first day (*P*=.16, partial η²=.06) or on the second day (*P*=.06, partial η²=.07). However, on the third day (*P*=.006, partial η²=.12) and fourth day (*P*=.005, partial η²=.12), the groups with physician support (DOC, APP+DOC) consumed significantly less oxycodone (Fig 3C). Specifically, APP+DOC consumed less than DOC on the second postoperative day (difference between APP+DOC and DOC: Cohen’s *d*=0.4).

All patients were prescribed 20 mg oxycodone daily, for a total of 80 mg oxycodone (Table 1). However, the average quantity of oxycodone consumption differed significantly between groups (*P*=.007, partial η²=.12; Fig 3C). Specifically, TIME consumed significantly more oxycodone than DOC (*P*=.005) and APP+DOC (*P*=.004), and TAU also consumed significantly more oxycodone than DOC (*P*=.02) and APP+DOC (*P*=.005). Moreover, APP+DOC showed lower total oxycodone consumption compared to DOC, with an effect size of Cohen’s d=0.2.

The groups differed significantly in their ratings for treatment expectation satisfaction (*P*=.04, partial *η²=*.10). Specifically, patients in APP+DOC were significantly more satisfied with their postoperative medication-related treatment and thought it was more successful than patients in TAU (*P*=.04).
