# Supplementary material for: A digital application and augmented physician rounds reduce postoperative pain and opioid consumption after primary total knee replacement (TKR): a randomized clinical trial
Source: BMC Med. 2022 Dec 5;20:469. doi: 10.1186/s12916-022-02638-0 (PMC9721029; doi:10.1186/s12916-022-02638-0)
Supplement: Supplementary file 2 — Additional file 2: Doc S2. Study Protocol (References: [58–70]). [file 12916_2022_2638_MOESM2_ESM.docx]

The supplement contains the following items:

1. Original protocol (p. 2-5)
2. Summary of changes for the original protocol (p. 6)
3. Final protocol (p. 7-11)
4. Original statistical analysis plan (p. 12-13)
5. Summary of changes for the statistical analysis plan (p. 14)
6. Final statistical analysis plan (p. 15-19)

# Original protocol

## Characteristics

- Study Type: Interventional
- Allocation: Randomization controlled trial
- Blinding: Blinded
- Who is blinded: patient/subject, investigator/therapist, assessor
- Control: Active control (effective treatment of control group)
- Purpose: Treatment
- Assignment: Parallel
- Off-label Drug use: no

## Health Condition or Problem studies

Knee replacement operation with standardized post-operative pain medication according to German guidelines

## Interventions

The interventions in short are listed here, for a more detailed description see section 1.6.4 Active treatment.

- Arm 1:   Treatment as Usual (TAU), pain medication according to the AWMF S3-guidelines
- Arm 2:   Hidden medication (standard treatment in hospital; dispenser with medication without extra information about content and mechanisms), pain medication according to the AWMF S3-guidelines, time-contingent (morning = 6am, midday = noon, evening = 6pm, night = midnight)
- Arm 3:   open medication 1 (Patient is informed about Medication s/he receives), pain medication according to the AWMF S3-guidelines plus optical and cues related to other senses (smell and haptic of medication) in regard to the medication
- Arm 4:   open medication 2 (Patient is informed about Medication s/he receives), pain medication according to the AWMF S3-guidelines plus optical and cues related to other senses (smell and haptic of medication) in regard to the medication, plus personal information delivery regarding pain medication
- Arm 5:   open medication 3, pain medication according to the AWMF S3-guidelines plus personal information delivery regarding pain medication

## Inclusion and Exclusion Criteria

### Inclusion Criteria

- Gender: male and female
- Minimum Age: 18 years
- Maximum Age: no maximum age
- Male and female participants that will undergo a knee joint replacement operation with standardized operation and standardized post-operative pain medication

### Exclusion Criteria

- Acute or chronic somatic disorders/pain and/or tumour/related pain, which need additional or special pain therapy
- Acute and chronic psychiatric disorders (according to DSM-IV)
- Insufficient German language knowledge
- Cognitive impairment
- Mind-altering medication

## Specific Study Design

The study design will consist of following steps, which will be explained in more detail in the following. In brief, patients will be screened on paper for eligibility; all eligible patients will be screened in person and the study will be comprehensively explained to them. Next, patients will be asked for consent. After providing written consent, the baseline data will be assessed via a standardized preoperative interview. After the assessment of the baseline data, patients will be randomized to one of the study group. After group allocation, patients will receive a preoperative questionnaire. One day after the surgery, patients will receive treatment depending on group allocation. After four days of treatment, patients will receive a postoperative questionnaire consisting of the same questionnaire as in the preoperative version and a conclusive interview will be performed to obtain information about treatment content, remarks and pain.

### Screening

Patients who will be planned to receive a TKR in the following week will be screened on paper, based on their medical history.

The study physician will check if the potential patients will fulfil the inclusion criteria (see inclusion/exclusion criteria). One day prior to the surgery, the study physician will screen the remaining eligible patients in person to verify if they fulfil the inclusion criteria. Further, the study physician will ask the patients, who were eligible for the study, if they are interested in participating in the study. If patients express their interest, a psychological researcher will explain the study in more detail to the patients and will ask for their consent.

### Method of treatment assignment

The treatment assignment will be randomized with stratification of gender and age. Patients who receive a TKR are usually 70 years old in average (18); therefore we will stratify the patients for over and under 70 years, so that the mean age and age distribution will not differ between groups. Further, approximately 60% of the patients who receive a TKR are female (19). Therefore, we intend that 60% of the patients in each group will be female and 40% of the patients will be male. Consequently, we plan to include 14 female and 10 male patients per group. We will continue with the recruitment phase until all groups include 20 patients and we will consider an even age distribution in the recruitment phase.

### Baseline

After the patients filled in written consent, a first interview will be performed. Subsequently, patients will be randomly assigned to their intended group and will receive a preoperative questionnaire which they will complete until the next day (day of surgery; for more details about specific questionnaires see SAP).

### Active treatment

The patients will receive the active treatment according to their group allocation for four postoperative days.

The groups will be as follows:

1. Treatment group (open medication; “APP”)

Patients in APP group will be supported by an iPad-based application, which will visualize medication effects through a medication dose-effect prognostic curve. Further, patients will receive acoustic and visual stimuli at times when they should take their analgesics, and will be provided with information about the pain medication. Further, the included medication dispenser will employ the same colors as the medication in the application.

2. Treatment group (open medication; “DOC”)

Patients in DOC group will receive additional postoperative visits by a patient-oriented and trained physician who will follow a half-structured questionnaire. The study physician will be a male physician who works in the orthopedic department at the corresponding German hospital center.

The approach of the physician will be as follows:

- The physician will create an environment of trust by emphasizing that he is part of the orthopedic team and that he will be their contact person. He will highlight the fact that he will be the responsible physician.
- The physician will further emphasize, that he will visit the patients every day for the next four days to answer their questions and support them.
- He will reassure the patients and will explain that he will do his best to support them.
- The physician’s half standardized questionnaire will consist of questions with regards to patients’ overall condition, mobility, pain, wound, improvement in physiotherapy, handling the pain diary, mood of the patient, atmosphere in the hospital room, analgesics, extent of swollen clinical local finding, and degree of movement.
- The patients will have the possibility to ask questions and express their concern.
- The expenditure will total to 5-10 minutes per patient for the first day and to 3-5 minutes for the remaining 3 days.

3. Treatment group (optimized open medication; “APP+DOC”)

Patients will be supported by the iPad application (see APP group) and the additional positive patient-oriented physician visits (see DOC group).

### Types of control

The type of controls will be two control groups:

1. The therapy as usual group (TAU) in which the patients will receive the standard care for TKR.
2. The time-controlled group in which the patients will receive a different medication dispenser for their analgesics. The modified dispenser will display concrete medication intake times (6am, noon, 6pm, and midnight). In contrast, the usual dispenser only refers to “morning”, “noon”, and “evening”.

### Postoperative questionnaires and conclusive interview

After four days following the TKR, patients will receive a postoperative questionnaire which correspondents to the preoperative questionnaire and a conclusive interview will be performed. Next, the patients will leave the hospitalized setting.

### Postoperative medical treatment

Medical treatment will be based on nation-wide guidelines and will therefore be as follows:

- Standard:
  - - 4x1 Metamizole 500 mg
    - 2x1 Celebrex 200mg
    - 2x1 Oxycodone 10 mg
    - If needed Morphine sulphate 10 mg (max. 6 per day)
- In case of Metamizole intolerance
  - - 4x2 Paracetamol 500 mg
    - 2x1 Celebrex 200 mg
    - 2x1 Oxycodone 10 mg
    - If needed Morphine sulphate 10 mg (max. 6 per day)
- In case of cardiac insufficiency
  - - 4x1 Metamizole 500 mg
    - 3x1 Ibuprofen 600 mg
    - 2x1 Oxycodone 10 mg
    - If needed Morphine sulphate 10 mg (max. 6 per day)
- In case of renal insufficiency
  - - 4x1 Metamizole 500 mg
    - 2x1 Oxycodone 10 mg
    - If needed Morphine sulphate 10 mg (max. 6 per day)

## Randomisation

Patients will be randomly assigned to one of the five groups. The randomization will be performed by an independent researcher who will have 10 piles of paper (pile 1-5 for all participating groups and for patients who are < 71 years and pile 6-10 for five different treatment groups for patients who are ≥ 71 years). The randomization will be stratified according to age and gender. Moreover, it will considered that the generally 60% of the patients who receive a TKR are female.

## Data handling

All data acquired will only be available to the principal investigators and employees involved in the data management. Data management will solely be performed with anonymous data that does not include any personal information about the subjects, but only previously assigned identification numbers. Also, publications will be based only on anonymous data.

# Summary of Changes

## Study process

There were no changes in the study process. The study could be performed as planned from the first included patient until the last included patient.

## Number of patients

The number of patients was altered from 100 patients in total to 120 patients in total due to a new power-calculation (see SAP).

## Recruitment start

The start of the recruitment started one day later as planned, because we waited until we received the registration confirmation.

# Final protocol

*General Study Design and Plan (in full length, see submitted SAP)*

## General study design

The study is a randomized, controlled trial with three treatment and two control groups, conducted at a German hospital center (Schoen Clinic Hamburg Eilbek).

### Level of blinding

The level of blinding depended on the stage of study. During the recruiting phase and first questionnaires, neither the recruiter nor the patient knew what group the patient was assigned to. This procedure minimized the possibility that patients decided to participate in the study based on their group allocation and to minimize the observer effect(20). Hence, all patients received information about all possible study related groups through a standardized information scheme and agreed to participate in the study before they were aware about their group allocation. After the first questionnaire, which covered the data for the baseline survey, the recruiter received the group allocation of the patient via text message from an independent researcher. Consequently, during the intervention, patients and study related researcher were aware which group the patient was assigned to. In contrast, the general hospital staff was not informed about the study participation to enable an unbiased general hospital care.

### Method of treatment assignment

The treatment assignment was randomized with stratification of gender and age. Patients who receive a TKR are usually 70 years old in average (18); therefore we stratified the patients for over and under 70 years, so that the mean age and age distribution does not differ between groups. Further, approximately 60% of the patients who received a TKR are female (19). Therefore, we intended that 60% of the patients in each group were female and 40% of the patients were male. Consequently, we planned to include 14 female and 10 male patients per group. We continued with the recruitment phase until all groups included 24 patients and we considered an even age distribution in the recruitment phase.

## Specific Study Design

The study design consisted of following steps (figure 1), which are explained in more detail in the following. In brief, patients were screened on paper for eligibility; all eligible patients were screened in person and the study was comprehensively explained to them. Next, patients were asked for consent. After providing written consent, the baseline data was assessed via a standardized preoperative interview, which consists of questions regarding demographic characteristics, pain, mobility and expected pain, and expected mobility. After the assessment of the baseline data, patients were randomized to a study group. After group allocation, patients received a preoperative questionnaire, which assessed pain related and well-being via von Korff(21) and SBI(22), self-instructions via FSS(23), Depression and Anxiety via PHQ-4(24), Lequesne index(25), and treatment expectations via SETS(26). One day after the surgery, patients receive treatment depending on group allocation. After four days of treatment, patients receive a postoperative questionnaire consisting of the same questionnaire as in the preoperative version and a conclusive interview was performed to obtain information about treatment content, remarks and pain.


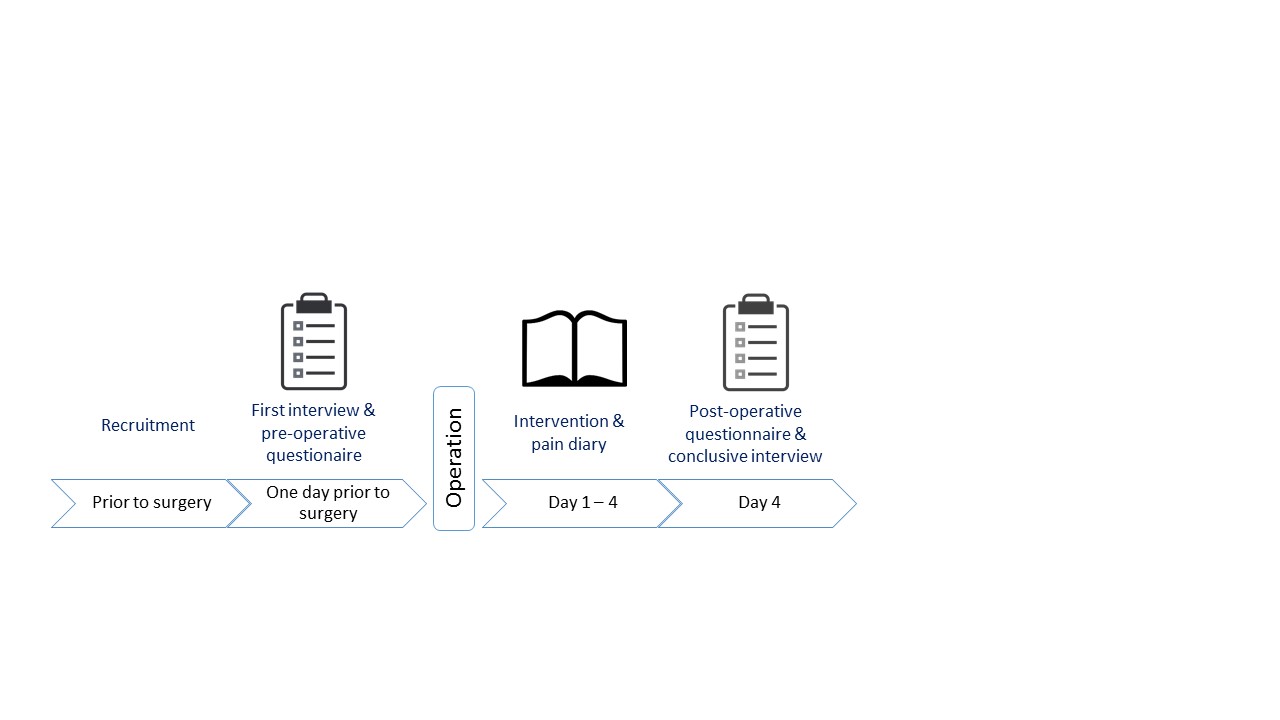


Figure 1. Study design

### Screening

Patients who were planned to receive a TKR in the following week were screened on paper, based on their medical history.

The study physician checked if the potential patients fulfilled the inclusion criteria (see inclusion-exclusion criteria). One day prior to the surgery, the study physician screened the remaining eligible patients in person to verify if they fulfilled the inclusion criteria. Further, the study physician asked the patients, who were eligible for the study, if they were interested in participating in the study. If patients expressed their interest, a psychological researcher explained the study in more detail to the patients and asked for their consent.

Inclusion-Exclusion Criteria

*Inclusion criteria:*

- - Patients who were admitted to a surgical ward at a hospital Center in Hamburg (Schoen clinics Hamburg Eilbek), Germany undergoing an elective and primary TKR
  - The TKR was performed due to knee osteoarthritis
  - Males or females
  - At least 18 years old
  - Voluntary participation

*Exclusion criteria:*

- - Acute or chronic somatic diseases/pain and/or tumor related pain, which required a special postoperative pain management other than the standardized management
  - Acute and/ or chronic mental disorders according to DSM-IV/ DSM-V
  - Insufficient written and/ or verbal German language skills
  - Cognitive impairment
  - Required or voluntarily intake of medication or substances, which alters state of consciousness (e.g., psychotropic or illegal drugs)
  - Patients who did not receive their analgesics in tablet forms but through an infusion pump

### Baseline

After the patients filled in written consent, a first interview was performed. Subsequently, patients were randomly assigned to their intended group and received a preoperative questionnaire which they completed until the next day (day of surgery).

### Active treatment

The patients received the active treatment according to their group allocation for four postoperative days.

The groups were as follows:

1. Treatment group (open medication; “APP”)

Patients in APP group were supported by an iPad-based application, which visualized medication effects through a medication dose-effect prognostic curve, had acoustic and visual stimuli at times when patients should take their analgesics, and were provided with information about the pain medication. Further, the included medication dispenser employed the same colors as the medication in the application.

2. Treatment group (open medication; “DOC”)

Patients in DOC group had additional postoperative visits by a patient-oriented and trained physician who followed a half-structured questionnaire. The study physician was a male physician who worked in the orthopedic department at the corresponding German hospital center.

The approach of the physician was as follows:

- The physician created an environment of trust by emphasizing that he is part of the orthopedic team and that he will be their contact person. He highlighted the fact that he is the responsible physician, because patients were often not aware about the functions of the treating person (e.g., physicians and nurses wear the same tunic in clinic where the study was performed).
- The physician further emphasized, that he will visit the patients every day for the next four days to answer their questions and support them.
- He reassured the patients and explained that he will do his best to support them.
- The physician’s half standardized questionnaire consisted of questions with regards to patients’ overall condition, mobility, pain, wound, improvement in physiotherapy, handling the pain diary, mood of the patient, atmosphere in the hospital room, analgesics, extent of swollen clinical local finding, and degree of movement.
- The patients had the possibility to ask questions and express their concern.
- The expenditure totaled to 5-10 minutes per patient for the first day and to 3-5 minutes for the remaining 3 days.

3. Treatment group (optimized open medication; “APP+DOC”)

Patients were supported by the iPad application (see APP group) and the additional positive patient-oriented physician visits (see DOC group).

### Types of control

The type of controls are two control groups:

1. The therapy as usual group (TAU) in which the patients received the standard care for TKR.
2. The time-controlled group in which the patients received a different medication dispenser for their analgesics. The modified dispenser displayed concrete medication intake times (6am, 12noon, 6pm, and 12midnight). In contrast, the usual dispenser only refers to “morning”, “noon”, and “evening”.

### Postoperative questionnaires and conclusive interview

After four days following the TKR, patients received a postoperative questionnaire which correspondents to the preoperative questionnaire and a conclusive interview was performed. Next, the patients left the hospitalized setting.

### Postoperative medical treatment

Medical treatment were based on nation-wide guidelines and were therefore as follows:

- Standard:
  - - 4x1 Metamizole 500 mg
    - 2x1 Celebrex 200mg
    - 2x1 Oxycodone 10 mg
    - If needed Morphine sulphate 10 mg (max. 6 per day)
- In case of Metamizole intolerance
  - - 4x2 Paracetamol 500 mg
    - 2x1 Celebrex 200 mg
    - 2x1 Oxycodone 10 mg
    - If needed Morphine sulphate 10 mg (max. 6 per day)
- In case of cardiac insufficiency
  - - 4x1 Metamizole 500 mg
    - 3x1 Ibuprofen 600 mg
    - 2x1 Oxycodone 10 mg
    - If needed Morphine sulphate 10 mg (max. 6 per day)
- In case of renal insufficiency
  - - 4x1 Metamizole 500 mg
    - 2x1 Oxycodone 10 mg
    - If needed Morphine sulphate 10 mg (max. 6 per day)

## Randomisation

Patients were randomly assigned to one of the five groups. The randomization was performed by an independent researcher who had 10 piles of paper (pile 1-5 for all participating groups and for patients who were < 71 years and pile 6-10 for five different treatment groups for patients who were ≥ 71 years). The randomization was stratified according to age and gender (see table 2). Due to the overall gender distribution of 60% female patients in a typical sample for a TKR surgery, we planned to include 14 women and 10 men per group. Further, due to the overall mean age of 70 years, we planned to include 10 patients who were 71 years of age and older and 14 patients who were maximal 70 years old per group.

Table 2. Planned patient distribution. It is planned to include 24 patients per group and to consider age and gender for the distribution. The amendment “+” stands for additional physician visits and/or application. Whereas the amendment “-“symbolizes no additional physician visits and/or application

|  | **App +** | | | | | **App -** | | | | |
| --- | --- | --- | --- | --- | --- | --- | --- | --- | --- | --- |
| **Doctor +** | Group APP+DOC = 24 | | | | | Group DOC = 24 | | | | |
|  |  | Men | | Women | |  | Men | | Women | |
|  | ≤ 70 | 6 | | 7 | | ≤ 70 | 6 | | 7 | |
|  | > 70 | 4 | | 7 | | > 71 | 4 | | 7 | |
| **Doctor -** | Group APP = 24 | | | | | Group TIME = 24  Group TAU = 24 | | | | |
|  |  | | Men | | Women |  | | Men | | Women |
|  | ≤ 70 | | 6 | | 7 | ≤ 70 | | 6 | | 7 |
|  | > 71 | | 4 | | 7 | > 71 | | 4 | | 7 |
|  | | | | | | | | | | |

# Original statistical Analyses plan

*See registration (DRKS)*

## Endpoints

*Primary endpoint*

Subjective ratings of the acute post-operative pain on a numerical rating scale from 0 (no pain) to 10 (worst pain imaginable), before operation and up to five days after operation; at least once a day.

*Secondary endpoints*

Physical condition (mobility), before operation and on day 4-5 after operation, measured on a numerical rating scale from 0 = full mobility to 100 = no mobility; pain medication consumption

## Target sample size

A power calculation with effects size = .02, α = .05 and Power = .95, five groups and 3 measurement times, G*Power(27) yielded a needed total sample size of 100 patients.

## Hypotheses

We hypothesize that APP+DOC shows the greatest effects, followed by APP and DOC, followed by TIME, with the least effect for TAU.

## Baseline Data

Baseline data to evaluate potential preoperative group differences will collected vial Interview and preoperative questionnaire.

The variables derived from the interview will be:

1. Demographic characteristics

- Gender on a nominal scale.
- Age on a ratio scale.

1. Preoperative pain. This variable is measured on a 0-10 numeric rating scale (NRS) for which are 0 = no pain and 10 = worst pain imaginable
2. Expected pain the first days after the surgery. This variable is measured on a 0-10 numeric rating scale (NRS) for which are 0 = no pain and 10 = worst pain imaginable.
3. Subjective physical functional impairment. This variable will be measured on a 0-10 numeric rating scale (NRS) for which are 0 = no functional impairment and 10 = complete functional impairment.
4. Expected subjective physical functional impairment after the surgery. This variable will be measured on a 0 – 10 numeric rating scale (0-10) for which are 0 = no functional impairment and 10 = complete functional impairment.

Variables from the preoperative questionnaire set will be:

1. Preoperatively acute knee pain on a NRS 0-10 (1 question).
2. Lequesne-Index captures pain (five questions), walking performance (two questions), and coping with everyday life (4 questions).
3. PHQ-4 questionnaire, which asses two questions concerning depression (PHQ-2) and two questions concerning generalized anxiety (GAD-2).
4. FSS – Questions related to pain related self-instructions. The questionnaire includes hindering and beneficial self-instructions and consists of 18 items.
5. Stanford Expectations of Treatment Scale (SETS) consists of six items which focus on the expectations of the patients towards their treatment outcome. The expectations are divided into positive and negative treatment expectation. We explore the treatment expectation respectively to the analgesics.

## Statistical analyses

*Statistical analyses*

The primary endpoint will be analyzed via repeated measures analyses of variance (rANOVA) with post-hoc tests corrected for multiple comparisons. The secondary outcome will be analyzed with via repeated measures analyses of variance with post-hoc tests corrected for multiple comparisons. Post-hoc analyses will be Fisher’s Least Significant Difference (LSD) post hoc test.

All necessary assumptions for ANOVA will be tested for, and statistical analyses will be adjusted, if necessary.

*Covariates*

For age and gender will be controlled for, if necessary.

*Multiple Testing*

To prevent accumulated α- errors, the analyses were Bonferroni corrected when necessary. Further, adequate post hoc tests were applied for all performed analyses.

## Missing Data

For the primary Outcome missing data will be analysed and completed with Last Observation Carried Forward (LOCF) method. For the secondary outcome, the missing will be not included.

# Summary of Changes

## Power Calculation

We rerun the power calculation before terminating the study and decided that based on primary outcome (pain relief), it would be advisable to use two number of measurements instead of three. The adjusted power analysis, calculated with G*Power (28), yielded a sample size of *N* = 120 patients for an expected small effect size of Cohen’s *d* = 0.2, two measurement time points, and a level of significance of α = 0.05. Therefore, we adjusted the target sample to 120 patients instead of 100 patients.

# Final Statistical Analyses Plan

*Statistical Analyses Plan (in full length, see submitted SAP)*

## Hypotheses

We hypothesize that APP+DOC shows the greatest effects, followed by APP and DOC, followed by TIME, with the least effect for TAU.

## Endpoints

We tested the application with regards to patient-related outcome measures.

*The primary outcome* is:

1. Postoperative pain measured in course of pain for four subsequent days following the surgery starting the day after the surgery and the pain relief indicated by the difference between pre- and postoperative pain.

*The secondary outcomes* are:

1. The quantity of analgesics (especially oxycodone).
2. Subjective functional capacity. (NOTE: This outcome is not included in the finalized article, due to limited word count on the one hand, and because in retrospect an analysis of either an objective functional capacity or another validated questionnaire would have been more informative)
3. Subjective treatment expectations interacting with subjective treatment success.

## Power Calculation:

The adjusted power analysis, calculated with G*Power (28), yielded a sample size of *N* = 120 patients for an expected small effect size of Cohen’s *d* = 0.2, two measurement time points, and a level of significance of α = 0.05.

## Study Variables

After patients gave their written informed consent to participate in the study, baseline data were collected. Baseline data were acquired through the first interview and included demographic information, preoperative chronic knee pain, expected pain, functional impairment, expected postoperative functional impairment, attitudes and experiences related to analgesics, and fear of surgery. As outcome variables, postoperative pain, analgesics consumption, subjective and objective functional capacity, and treatment success were evaluated.

*Baseline*

Subsequently, the specific variables in the interview were:

1. Demographic characteristics

- Gender on a nominal scale.
- Age on a ratio scale.

1. Preoperative pain. This variable is measured on a 0-10 numeric rating scale (NRS) for which are 0 = no pain and 10 = worst pain imaginable.
2. Expected pain the first days after the surgery. This variable is measured on a 0-10 numeric rating scale (NRS) for which are 0 = no pain and 10 = worst pain imaginable.
3. Subjective physical functional impairment. This variable is measured on a 0-10 numeric rating scale (NRS) for which are 0 = no functional impairment and 10 = complete functional impairment.
4. Expected subjective physical functional impairment after the surgery. This variable is measured on a 0 – 10 numeric rating scale (0-10) for which are 0 = no functional impairment and 10 = complete functional impairment.

Moreover, after the first interview, patients received a preoperative questionnaire set. The patients had time until their surgery (one day) to complete the questionnaires. This set includes:

1. Preoperatively acute knee pain on a NRS 0-10 (1 question).
2. Lequesne-Index captures pain (five questions), walking performance (two questions), and coping with everyday life (4 questions).
3. PHQ-4 questionnaire, which asses two questions concerning depression (PHQ-2) and two questions concerning generalized anxiety (GAD-2).
4. FSS – Questions related to pain related self-instructions. The questionnaire includes hindering and beneficial self-instructions and consists of 18 items.
5. Stanford Expectations of Treatment Scale (SETS) consists of six items which focus on the expectations of the patients towards their treatment outcome. The expectations are divided into positive and negative treatment expectation. We explore the treatment expectation respectively to the analgesics.

### Outcomes

The analyses for the outcome variable differed and depended on the investigated variable and are described individually.

*Primary Outcome*

1. The course of postoperative pain. After the surgery, patients received a pain diary which asked for the pain rate on a NRS 0-10 of the patients every 2 hours starting 6am until midnight for four days, starting the first day after the surgery.

- Analysis: The course of pain will be analyzed with the repeated measures ANOVA (rANOVA). The pain level was measured 4 times a day (within) and the groups will be the between-factor. To compare the days separately, the course of pain for the individual days was analyzed between the groups. Fisher’s Least Significant Difference (LSD) post hoc test was applied to explore the differences in more detail.

1. Comparison of pre- and postoperative pain. The patients were asked about their pain the day prior to their surgery and four days after the surgery on a NRS 0-10. To detect the pain relief, we calculated the difference between pre- and postoperative pain and compared the number of patients who experienced a pain reduction between the groups. Further, the differences of extent of pain reduction between the groups was explored.

- Analysis: A difference value was calculated between pre- and postoperative pain. This value was analyzed with ANOVA to explore group differences. Fisher’s Least Significant Difference (LSD) post hoc test was applied to explore the differences in more detail.

*Secondary Outcome*

1. The analgesic consumption was explored. Patients received their analgesics in according with guidelines from the medical professional society (AWMF). The prescription of analgesic was documented by medical care takers and patients. The administration of analgesics depended on pre-existing conditions.

- Analysis: Independent of pre-existing conditions, all patients had a prescription of 20mg oxycodone daily. It was analysed how much oxycodone patients received after four days in total and for four days respectively. Further, the differences were analysed with the Fisher’s Least Significant Difference (LSD) post hoc test. (NOTE: to differentiate the effects between two significant groups the effect size Cohen’s d will be calculated.)

1. Subjective physical functional impairment. This variable is measured on a 0-10 numeric rating scale (NRS) for which are 0 = no physical functional impairment and 10 = complete physical functional impairment.

- Analysis: The subjective physical functional impairment is analysed with repeated measured ANOVA. Preoperative and postoperative functional impairment were used as reference times (within) for groups (between). (NOTE: This outcome was not included into the finalized article).

1. Subjective treatment success. Patients complete the SETS four days subsequent the surgery to analyze the group differences of treatment success compared to the treatment expectations.

- Analysis: the treatment success is analysed with SETS, using the rANOVA. Negative and positive subjective expectation/success was explored by applying treatment expectations and treatment success as reference times (within) for the groups (between). Significant results were further analysed with LSD post hoc test.

## Sample Size

The power analysis, calculated with G*Power(28) determined, that a sample size of *N* = 120 patients were required for an expected small to medium effect size of Cohen’s *d* = 0.2, for two time of measurements, and a level of significance of α = 0.05. Thus, we planned to include 120 patients, i.e., 24 patients per group.

## General Considerations

### Analysis Populations

The population which are included into the analyses were the patients who fulfilled the inclusion and did not fulfil the exclusion criteria, gave written consent, and participated in the study. Further, patients were only included if they completed at least one post-baseline assessment or questionnaire.

For each analysis, patients were only included if they provided a complete data set, which was necessary to evaluate relevant calculated analysis (excluded case pairwise).

In case, there would have been a relevant amount of incomplete data, patients which did not provide complete data, were analyzed and compared to patients who provided complete data.

### Covariates and Subgroups

Based on the study design and pre-studies, no covariates were absolutely necessary. We assumed that due to the reason that we used digital media and younger patients could be more accustomed to the usage of digital media, age could be an influential factor. However, we controlled for age by using stratified randomization. Nevertheless, we identified age as covariate and planned to control for it, if it seems to be necessary. The applied model therefore is the forward stepwise selection. Covariate analyses revealed that neither age nor gender was a covariate and had especially be controlled for.

### Missing Data

Missing values were handled depending on the outcome variable. The data for the primary outcome were derived by entries in pain diary on NRS 0-10 (0 = no pain, 10 = worst pain imaginable). Patients stated that they often entered a pain value if the pain value changed and did not fill in a pain value if the pain value stayed consistent. Therefore, the missing values was completed with Last Observation Carried Forward (LOCF) method.

For the course of pain several missing values had to be replaced. Patients filled in the pain diary for every two hours. However, for the analysis only pain entries at four distinct time points (6am, 12noon, 6pm, and 12midnight) were analyzed. Nevertheless, the not analyzed pain entries could be used to replace missing values, e.g. some patients slept already at midnight and stated their pain at 10pm the last time for certain days.

Further, for the secondary outcomes, missing data were not replaced, because it is assumed that the variables are missing at random. Further, only complete data sets were used for further analysis.

### Significance

Two-sided p-values of *p* = .05 or less were considered to indicate statistical significance.

### Multiple Testing

To prevent accumulated α- errors, the analyses were Bonferroni corrected when necessary. Further, adequate post hoc tests were applied for all performed analyses.

### Analyses of Assumptions

*Primary Outcome*

1. Pain: Course of pain, pain relief

Due to sample size (n_i_ = 24 and therefore > 10), normality did not have to be controlled for. However, Q-Q plots confirmed the normal distribution of the primary outcome. For the course of pain, the assumption of sphericity was violated, therefore the degrees of freedom will be Greenhouse-Geisser corrected (ε = 0.46). For pain relief, sphericity test was not necessary.

*Secondary Outcome*

1. Treatment Expectation

Due to sample size (n_i_ = 24 and therefore > 10), normality did not have to be controlled for. However, Q-Q plots confirmed the normal distribution. Due to only two time of measurements, the analysis for treatment expectation does not to be checked for sphericity.

1. Self-reported restriction on mobility

Due to sample size (n_i_ = 24 and therefore > 10), normality did not have to be controlled for. Due to only two time of measurements, the analysis for SETS did not have to be checked for sphericity.

1. Oxycodone consumption

Due to sample size (n_i_ = 24 and therefore > 10), normality did not have to be controlled for. For oxycodone consumption, the assumption of sphericity was violated, therefore the degrees of freedom were Greenhouse-Geisser corrected (ε = 0.61).

## Reporting Conventions

P-values ≥.001 to .01 were reported to 3 decimal places; p-values less than 0.001 were reported as “<0.001”; p-values >.01 were reported to 2 decimal places. The mean, standard deviation, and any other statistics other than quantiles, were reported to two decimal places, except in baseline characteristics in which they were portrait without decimal places.

## Technical Details

The analyses were made with SPSS 25.0 on a Windows Computer. The code was saved as a Syntax to be comprehensible in retrospect. The analyses were performed by one of the psychological researchers. Further, at the beginning of the syntaxes, there is a comment which includes the time, date, author, references to input, and output data.

**6.9 Note: Report of data**

To investigate the additive and synergistic effects of digital health and augmented physician rounds, we implemented a 2x2 full factorial study design (Fig 1). This study design is thus far the only possibility to examine the effects of two new interventions (APP and DOC) separately and in their interaction (APP+DOC) in comparison to the standard procedure (TAU).

Moreover, we investigated another group (TIME) to control for a potential bias effect deriving from the circumstance that the app reminded the patients to take their medication at exact time points. In this group, the patients also received their medications on a time-contingent basis but without APP and DOC. Since this group did not differ from the TAU group and had no influence on the results, we did not include it in our presentation of the results in favor of a more concise presentation of the study. However, the results including the group “TIME” can be found in the appendix of our manuscript.
