# Supplementary material for: A digital application and augmented physician rounds reduce postoperative pain and opioid consumption after primary total knee replacement (TKR): a randomized clinical trial
Source: BMC Med. 2022 Dec 5;20:469. doi: 10.1186/s12916-022-02638-0 (PMC9721029; doi:10.1186/s12916-022-02638-0)
Supplement: Supplementary file 3 — Additional file 3: Doc S3. Statistical Analysis Plan (References: [58–70]). [file 12916_2022_2638_MOESM3_ESM.docx]

Statistical Analysis Plan

| TRIAL FULL TITLE | App and physician visits reduce postoperative pain and opioid consumption |
| --- | --- |
| TRIAL REGISTRATION | DRKS German clinical trial registration number, DRKS00009554 |
| SAP VERSION DATE | 22.12.2020 |
| TRIAL STATISTICIAN | Julia Stuhlreyer |
| TRIAL CHIEF INVESTIGATOR | PD Dr. Regine Klinger and Julia Stuhlreyer |
| SAP AUTHOR | Julia Stuhlreyer |

# Table of Contents

[1 SAP Signatures 1](#_Toc42854054)

[2 Table of Contents 1](#_Toc42854055)

[3 Abbreviations and Definitions 3](#_Toc42854056)

[4 Introduction 3](#_Toc42854057)

[4.1 Preface 4](#_Toc42854058)

[4.2 Purpose of the analyses 4](#_Toc42854059)

[5 Study Objectives and Endpoints 4](#_Toc42854060)

[5.1 Study Objectives 4](#_Toc42854061)

[5.2 Endpoints 4](#_Toc42854062)

[Study Methods 5](#_Toc42854063)

[5.3 General Study Design and Plan 5](#_Toc42854064)

[5.4 Randomisation and Blinding 8](#_Toc42854065)

[5.5 Study Variables 8](#_Toc42854066)

[6 Sample Size 12](#_Toc42854067)

[7 General Considerations 12](#_Toc42854068)

[7.1 Timing of Analyses 12](#_Toc42854069)

[7.2 Analysis Populations 12](#_Toc42854070)

[7.3 Covariates and Subgroups 13](#_Toc42854071)

[7.4 Missing Data 13](#_Toc42854072)

[7.4.1 Stopping Rules 13](#_Toc42854073)

[7.4.2 Significance 13](#_Toc42854074)

[7.5 Multiple Testing 13](#_Toc42854075)

[8 Summary of Study Data 13](#_Toc42854076)

[8.1 Subject Disposition 13](#_Toc42854077)

[8.2 Protocol Deviations 14](#_Toc42854078)

[8.3 Demographic and Baseline Variables 14](#_Toc42854079)

[8.4 Concurrent Illnesses and Medical Conditions 14](#_Toc42854080)

[8.5 Prior and Concurrent Medications 14](#_Toc42854081)

[8.6 Treatment Compliance 14](#_Toc42854082)

[9 Efficacy Analyses 14](#_Toc42854083)

[9.1 Analyses of Assumptions 15](#_Toc42854084)

[9.2 Primary Efficacy Analysis 16](#_Toc42854085)

[9.3 Secondary Efficacy Analyses 16](#_Toc42854086)

[10 Safety Analyses 16](#_Toc42854087)

[10.1 Extent of Exposure 16](#_Toc42854088)

[10.2 Adverse Events 16](#_Toc42854089)

[10.3 Deaths, Serious Adverse Events and other Significant Adverse Events 16](#_Toc42854090)

[10.4 Pregnancies 16](#_Toc42854091)

[10.5 Other Safety Measures 16](#_Toc42854092)

[11 Figures and Tables 17](#_Toc42854093)

[12 Reporting Conventions 17](#_Toc42854094)

[13 Technical Details 17](#_Toc42854095)

[15 Listing of Tables, Listings and Figures 19](#_Toc42854097)

[15.1 Tables 19](#_Toc42854098)

[15.2 Figures 21](#_Toc42854099)

[15.3 Listing Tables and Figures 26](#_Toc42854100)

# Abbreviations and Definitions

| APP | iPad Application Group |
| --- | --- |
| APP+DOC | iPad Application and physician group |
| DOC | Physician group |
| LSD | Fisher’s Least Significant Difference (post hoc test) |
| NRS | Numeric Rating Scale |
| rANOVA | ANOVA repeated measures |
| SAP | **Statistical Analysis Plan** |
| SET | Stanford Expectations Treatment Scale (Questionnaire) |
| TAU | Therapy as Usual |
| TIME | Time controlled group |
| TKR | Total Knee Replacement |

# Introduction

## Preface

Medical guidelines for postoperative pain treatment schedule for all patients the same analgesics depending on the surgery and known pre-illnesses. However, recent research revealed that postoperative acute pain differs within and between patients and depend on contextual factors. Specifically, despite the same usual care after a knee replacement (TKR), 30% of the patients still suffer from severe postoperative and 20% of the patients still suffer from severe pain one year after the surgery (1). The differences in pain can partly be explained through patients’ thoughts, evaluations, and experiences. Another important influential factors are expectations. The effect of expectation as a context factor is especially confirmed in the open-hidden medication paradigm (11, 10). The open-hidden medication paradigm implicates that patients who receive their medication through a medical care taker (“open medication”) and are informed about their analgesics have a greater pain relief than patients who receive their medication through an infusion pump (“hidden medication”)(11, 31, 12). Moreover, the physician-patient relationship is an important context factor, e.g., patients who receive their physicians as empathic are more compliant towards their treatment (32).

Severe postoperative pain leads to a decreased treatment satisfaction and to an over prescription of opioids resulting in considerable health concerns (1). Given the opioid crisis (3), which is based on opioid misuse and addiction, the prescription of oxycodone must be minimized. Commonly, the overuse of opioids starts in the phase immediately after surgery (7). However, there are only few adequate alternatives to oxycodone, making its omission difficult (8). Currently, there is no clear guideline or concept how to reduce postoperative oxycodone prescription without increasing postoperative pain (9).

With the technological upswing, different application possibilities have been invented, but rarely been scientifically tested (33). Interestingly, the new possibilities given through digitalization combined with scientific findings have not been implemented for postoperative pain treatment so far. Thus, we developed an iPad-based application, which employs open medication, gives acoustic, and visual stimuli, and integrates the patient into the treatment. We tested the effectiveness of the App on patients, who received a TKR.

## Purpose of the analyses

The analyses assessed the efficacy of digitalization in regard to medication effect and patient engagement either with, without or solely regular contact (about 5 minutes) to a patient-oriented physician. The effects were compared to therapy as usual and a medication intake time-controlled group with regard to postoperative pain, analgesics intake, physical functional capacity, and subjective treatment success based on expectations.

# Study Objectives and Endpoints

## Study Objectives

The overall aim of this study is to minimize the postoperative acute knee pain and to decrease the need for analgesics, especially oxycodone. To achieve this objective, we included and applied recent research findings. Hence, we applied insights about medication effects and used a digital device in order to implement these insights. In particular, we applied visual and acoustic cues to transfer and depict the individual medication intake times, effects, and modes of action. Hereby, we attempted to establish an expectation effect related to subjective treatment success. We investigated the effects with regards to postoperative pain, medication intake, physical functional capacity, and treatment success.

## Endpoints

We tested the application with regards to patient-related outcome measures.

The primary outcome is pain:

1. Postoperative pain measured in course of pain for four subsequent days following the surgery starting the day after the surgery and the pain relief indicated by the difference between pre- and postoperative pain.

The secondary outcomes are:

1. The quantity of analgesics (especially oxycodone).
2. Subjective functional capacity. (NOTE: This is outcome is not included in the finalized article, due to limited word count on the one hand, and because in retrospect an analysis of either an objective functional capacity or another validated questionnaire would have been more informative)
3. Subjective treatment expectations interacting with subjective treatment success.

# Study Methods

## General Study Design and Plan

*Study design*

The study is a randomized, controlled trial with three treatment and two control groups, conducted at a German hospital center (Schoen Clinic Hamburg Eilbek).

*Types of control*

The type of controls are two control groups:

1. The therapy as usual group (TAU) in which the patients received the standard care for TKR.
2. The time-controlled group in which the patients received a different medication dispenser for their analgesics. The modified dispenser displayed concrete medication intake times (6am, 12noon, 6pm, and 12midnight). In contrast, the usual dispenser only refers to “morning”, “noon”, “evening”.

*Level of blinding*

The level of blinding depended on the stage of study. During the recruiting phase and first questionnaires, neither the recruiter nor the patient knew what group the patient was assigned to. This procedure minimized the possibility that patients decided to participate in the study based on their group allocation and to minimize the observer effect. Hence, all patients received information about all possible study related groups through a standardized information scheme and agreed to participate in the study before they were aware about their group allocation. After the first questionnaire, which covered the data for the baseline survey, the recruiter received the group allocation of the patient via text message from an independent researcher. Consequently, during the intervention, patients and study related researcher were aware which group the patient was assigned to. In contrast, the general hospital staff was not informed about the study participation to enable an unbiased general hospital care.

*Method of treatment assignment*

The treatment assignment was randomized with stratification of gender and age. Patients who receive a TKR are usually 70 years old in average (18); therefore we stratified the patients for over and under 70 years, so that the mean age and age distribution does not differ between groups. Further, approximately 60% of the patients who received a TKR are female (19). Therefore, we intended that 60% of the patients in each group were female and 40% of the patients were male. Consequently, we planned to include 14 female and 10 male patients per group. We continued with the recruitment phase until all groups included 24 patients and we considered an even age distribution in the recruitment phase (see table 1).

*Screening*

Patients who were planned to receive a TKR in the following week were screened on paper, based on their medical history.

The study physician checked if the potential patients fulfilled the inclusion criteria (see section 5.4). One day prior to the surgery, the study physician screened the remaining eligible patients in person to verify if they fulfilled the inclusion criteria. Further, the study physician asked the patients, who were eligible for the study, if they were interested in participating in the study. If patients expressed their interest, a psychological researcher explained the study in more detail to the patients and asked for their consent.

*Baseline*

After the patients filled in written consent, a first interview was performed. Subsequently, patients were randomly assigned to their intended group and received a preoperative questionnaire which they complete until the next day (day of surgery).

*Active treatment*

The patients received the active treatment according to their group allocation for four postoperative days.

The groups were as follows:

1. Treatment group (open medication; “APP”)

Patients in APP group were supported by an iPad-based application, which visualized medication effects through a medication dose-effect prognostic curve, had acoustic and visual stimuli at times when patients should take their analgesics, and were provided with information about the pain medication. Further, the included medication dispenser employed the same colors as the medication in the application (see visual portray of the application, page 29).

2. Treatment group (open medication; “DOC”)

Patients in DOC group had additional postoperative visits by a patient-oriented and trained physician who followed a half-structured questionnaire. The study physician was a male physician who worked in the orthopedic department at the corresponding German hospital center.

The approach of the physician was as follows:

- The physician created an environment of trust by emphasizing that he is part of the orthopedic team and that he will be their contact person. He highlighted the fact that he is the responsible physician, because patients were often not aware about the functions of the treating person (e.g., physicians and nurses wear the same tunic in clinic where the study was performed).
- The physician further emphasized, that he will visit the patients every day for the next four days to answer their questions and support them.
- He reassured the patients and explained that he will do his best to support them.
- The physician’s half standardized questionnaire consisted of questions with regards to patients’ overall condition, mobility, pain, wound, improvement in physiotherapy, handling the pain diary, mood of the patient, atmosphere in the hospital room, analgesics, extent of swollen clinical local finding, and degree of movement.
- The patients had the possibility to ask questions and express their concern.
- The expenditure totaled to 5-10 minutes per patient for the first day and to 3-5 minutes for the remaining 3 days.

3. Treatment group (optimized open medication; “APP+DOC”)

Patients were supported by the iPad application (see APP group) and the additional positive patient-oriented physician visits (see DOC group).

After four days following the TKR, the patients left the hospitalized setting.


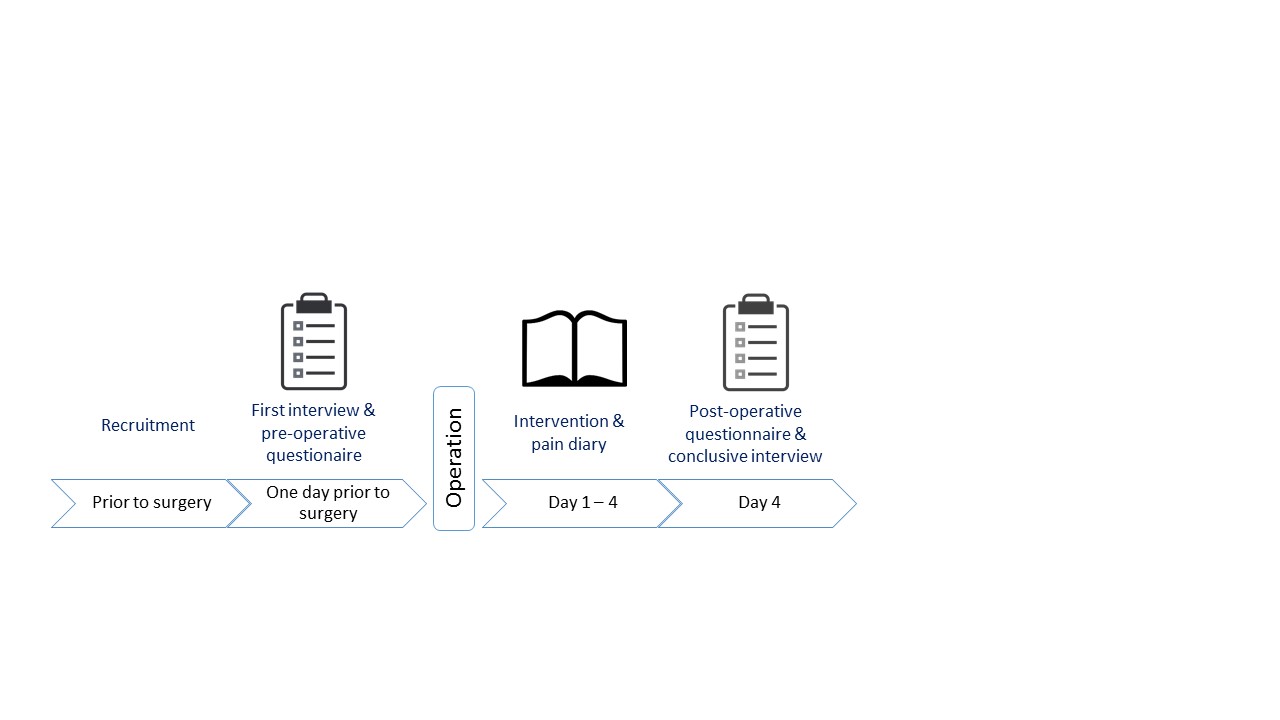


Figure 1. Study design

Inclusion-Exclusion Criteria and General Study Population

*Inclusion criteria:*

- - Patients who were admitted to a surgical ward at a hospital Center in Hamburg (Schoen clinics Hamburg Eilbek), Germany undergoing an elective and primary TKR
  - The TKR was performed due to knee osteoarthritis
  - Males or females
  - At least 18 years old
  - Voluntary participation

*Exclusion criteria:*

- - Acute or chronic somatic diseases/pain and/or tumor related pain, which required a special postoperative pain management other than the standardized management
  - Acute and/ or chronic mental disorders according to DSM-IV/ DSM-V
  - Insufficient written and/ or verbal German language skills
  - Cognitive impairment
  - Required or voluntarily intake of medication or substances, which alters state of consciousness (e.g., psychotropic or illegal drugs)
  - Patients who did not receive their analgesics in tablet forms but through an infusion pump

## Randomisation and Blinding

Patients were randomly assigned to one of the five groups. The randomization was performed by an independent researcher who had 10 piles of paper (pile 1-5 for all participating groups and for patients who were < 71 years and pile 6-10 for five different treatment groups for patients who were ≥ 71 years). The randomization was stratified according to age and gender (see Table 1). Due to the overall gender distribution of 60% female patients in a typical sample for a TKR surgery, we planned to include 14 women and 10 men per group. Further, due to the overall mean age of 70 years, we planned to include 10 patients who were 71 years of age and older and 14 patients who were maximal 70 years old per group.

Table 1. Planned patient distribution. It is planned to include 24 patients per group and to consider age and gender for the distribution. The amendment “+” stands for additional physician visits and/or application. Whereas the amendment “-“symbolizes no additional physician visits and/or application

|  | **App +** | | | | | **App -** | | | | |
| --- | --- | --- | --- | --- | --- | --- | --- | --- | --- | --- |
| **Doctor +** | Group APP+DOC = 24 | | | | | Group DOC = 24 | | | | |
|  |  | Men | | Women | |  | Men | | Women | |
|  | ≤ 70 | 6 | | 7 | | ≤ 70 | 6 | | 7 | |
|  | > 70 | 4 | | 7 | | > 71 | 4 | | 7 | |
| **Doctor -** | Group APP = 24 | | | | | Group TIME = 24  Group TAU = 24 | | | | |
|  |  | | Men | | Women |  | | Men | | Women |
|  | ≤ 70 | | 6 | | 7 | ≤ 70 | | 6 | | 7 |
|  | > 71 | | 4 | | 7 | > 71 | | 4 | | 7 |
|  | | | | | | | | | | |

## Study Variables

After patients gave their written informed consent to participate in the study, baseline data were collected. Baseline data were acquired through the first interview and included demographic information, preoperative chronic knee pain, expected pain, functional impairment, expected postoperative functional impairment, attitudes and experiences related to analgesics, and fear of surgery. As outcome variables, postoperative pain, analgesics consumption, subjective and objective functional capacity, and treatment success were evaluated (see Table 2).

*Baseline*

Subsequently, the specific variables in the interview were:

1. Demographic Information

- Gender on a nominal scale.
- Age on a ratio scale.

1. Preoperative pain. This variable is measured on a 0-10 numeric rating scale (NRS) for which are 0 = no pain and 10 = worst pain imaginable
2. Expected pain the first days after the surgery. This variable is measured on a 0-10 numeric rating scale (NRS) for which are 0 = no pain and 10 = worst pain imaginable.
3. Subjective physical functional impairment. This variable is measured on a 0-10 numeric rating scale (NRS) for which are 0 = no functional impairment and 10 = complete functional impairment.
4. Expected subjective physical functional impairment after the surgery. This variable is measured on a 0 – 10 numeric rating scale (0-10) for which are 0 = no functional impairment and 10 = complete functional impairment.

Moreover, after the first interview, patients received a preoperative questionnaire set. The patients had time until their surgery (one day) to complete the questionnaires. This set includes:

1. Preoperatively acute knee pain on a NRS 0-10 (1 question).
2. Lequesne-Index captures pain (five questions), walking performance (two questions), and coping with everyday life (4 questions).
3. PHQ-4 questionnaire, which asses two questions concerning depression (PHQ-2) and two questions concerning generalized anxiety (GAD-2).
4. FSS – Questions related to pain related self-instructions. The questionnaire includes hindering and beneficial self-instructions and consists of 18 items.
5. Stanford Expectations of Treatment Scale (SETS) consists of six items which focus on the expectations of the patients towards their treatment outcome. The expectations are divided into positive and negative treatment expectation. We explore the treatment expectation respectively to the analgesics.

*Outcome*

The analyses for the outcome variable differed and depended on the investigated variable and are described individually.

*Primary Outcome*

1. The course of postoperative pain. After the surgery, patients received a pain diary which asked for the pain rate on a NRS 0-10 of the patients every 2 hours starting 6am until 12midnight for four days, starting the first day after the surgery.

- Analysis: The course of pain will be analysed with the repeated measures ANOVA (rANOVA). The pain level was measured 4 times a day (within) and the groups will be the between-factor. To compare the days separately, the course of pain for the individual days was analysed between the groups. Fisher’s Least Significant Difference (LSD) post hoc test was applied to explore the differences in more detail.

1. Comparison of pre- and postoperative pain. The patients were asked about their pain the day prior to their surgery and four days after the surgery on a NRS 0-10. To detect the pain relief, we calculated the difference between pre- and postoperative pain and compared the number of patients who experienced a pain reduction between the groups. Further, the differences of extent of pain reduction between the groups was explored.

- Analysis: A difference value was calculated between pre- and postoperative pain. This value was analysed with ANOVA to explore group differences. Fisher’s Least Significant Difference (LSD) post hoc test was applied to explore the differences in more detail.

*Secondary Outcome*

1. The analgesic consumption was explored. Patients received their analgesics in according with guidelines from the medical professional society (AWMF). The prescription of analgesic was documented by medical care takers and patients. The administration of analgesics depended on pre-existing conditions. The analgesics’ options are:

- Standard:
  - - 4x1 Metamizole 500 mg
    - 2x1 Celebrex 200mg
    - 2x1 Oxycodone 10 mg
    - If needed Morphine sulphate 10 mg (max. 6 per day)
- In case of Metamizole intolerance
  - - 4x2 Paracetamol 500 mg
    - 2x1 Celebrex 200 mg
    - 2x1 Oxycodone 10 mg
    - If needed Morphine sulphate 10 mg (max. 6 per day)
- In case of cardiac insufficiency
  - - 4x1 Metamizole 500 mg
    - 3x1 Ibuprofen 600 mg
    - 2x1 Oxycodone 10 mg
    - If needed Morphine sulphate 10 mg (max. 6 per day)
- In case of renal insufficiency
  - - 4x1 Metamizole 500 mg
    - 2x1 Oxycodone 10 mg
    - If needed Morphine sulphate 10 mg (max. 6 per day)
- Analysis: Independent of pre-existing conditions, all patients had a prescription of 20mg oxycodone daily. It was analysed how much oxycodone patients received after four days in total and for four days respectively. Further, the differences were analysed with the Fisher’s Least Significant Difference (LSD) post hoc test. (NOTE: to differentiate the effects between two significant groups the effect size Cohen’s d will be calculated.)

1. Subjective physical functional impairment. This variable is measured on a 0-10 numeric rating scale (NRS) for which are 0 = no physical functional impairment and 10 = complete physical functional impairment.

- Analysis: The subjective physical functional impairment is analysed with repeated measured ANOVA. Preoperative and postoperative functional impairment were used as reference times (within) for groups (between). (NOTE: This outcome was not included into the finalized article).

1. Subjective treatment success. Patients complete the SETS four days subsequent the surgery to analyse the group differences of treatment success compared to the treatment expectations.

- Analysis: the treatment success is analysed with SETS, using the rANOVA. Negative and positive subjective expectation/success was explored by applying treatment expectations and treatment success as reference times (within) for the groups (between). Significant results were further analysed with LSD post hoc test.

*Table 2.* A summary display of collected data.

|  | Baseline | Day 1 – 4  Pain Diary | Day 4 (Day of Hospital discharge) |
| --- | --- | --- | --- |
| Demographics | x |  |  |
| Acute knee pain (NRS 0-10) | x | x | x |
| Expected Pain (NRS 0-10) | x |  |  |
| Functional impairment (NRS 0-10) | x |  | x |
| Expected functional impairment (NRS 0-10) | x |  |  |
| Attitudes related to analgesics | x |  |  |
| Experiences with analgesics | x |  |  |
| SETS questionnaire | x |  | x |
| Chronic and acute knee pain | x |  |  |
| Medication intake | x | x | x |

# Sample Size

The power analysis, calculated with G*Power(28) determined, that a sample size of *N* = 120 patients were required for an expected small to medium effect size of Cohen’s *d* = 0.2, for two time of measurements, and a level of significance of α = 0.05. Thus, we planned to include 120 patients, i.e., 24 patients per group.

# General Considerations

## Timing of Analyses

The recruitment phase was terminated when all five groups had at least 24 patients included. All data was obtained paper-based and not digitally. Subsequently, all questionnaires and pain diaries, were transferred into a SPSS file. Then the final analysis was performed.

## Analysis Populations

The population which are included into the analyses were the patients who fulfilled the inclusion and did not fulfil the exclusion criteria, gave written consent, and participated in the study. Further, patients were only included if they completed at least one post-baseline assessment or questionnaire.

For each analysis, patients were only included if they provided a complete data set, which was necessary to evaluate relevant calculated analysis (excluded case pairwise).

In case, there would have been a relevant amount of incomplete data, patients which did not provide complete data, were analysed and compared to patients who provided complete data.

Patients included in the analysis respectively

Table 2. Number of patients in- and excluded.

|  | Inclusion | Exclusion | Reason |
| --- | --- | --- | --- |
| Course of postoperative pain (LOCF) | 116 | 4 | Did not fill in the pain diary |
| Pain difference “before and after” (LOCF) | 120 | 0 |  |
| Oxycodone consumption | 120 | 0 |  |
| Treatment expectations and treatment success | 101 | 19 | Did not fill in expectations or treatment success |

## Covariates and Subgroups

Based on the study design and pre-studies, no covariates were absolutely necessary. We assumed that due to the reason that we used digital media and younger patients could be more accustomed to the usage of digital media, age could be an influential factor. However, we controlled for age by using block randomisation. Nevertheless, we identified age as covariate and planned to control for it, if it seems to be necessary. The applied model therefore is the forward stepwise selection. Covariate analyses revealed that neither age nor gender was a covariate and had especially be controlled for.

## Missing Data

Missing values were handled depending on the outcome variable. The data for the primary outcome were derived by entries in pain diary on NRS 0-10 (0 = no pain, 10 = worst pain imaginable). Patients stated that they often entered a pain value if the pain value changed and did not fill in a pain value if the pain value stayed consistent. Therefore, the missing values was completed with Last Observation Carried Forward (LOCF) method.

For the course of pain several missing values had to be replaced. Patients filled in the pain diary for every two hours. However, for the analysis only pain entries at four distinct time points (6am, 12noon, 6pm, and 12midnight) were analysed. Nevertheless, the not analysed pain entries could be used to replace missing values, e.g. some patients slept already at midnight and stated their pain at 10pm the last time for certain days. The following table gives insight about the replaced missing values through LOCF.

Table 3. Replaced missing value for course of pain

|  |  |  |  | Replaced for | | | | |
| --- | --- | --- | --- | --- | --- | --- | --- | --- |
|  | Missing values | Replacements | Not replaced | Group1 | Group2 | Group3 | Group4 | Group5 |
| Day1 6am | 34 | Night day1 =34 | 0 | 7 | 3 | 5 | 9 | 10 |
| Day1 noon | 18 | Night day 1=2  6am=1  8am=1  9am=1  10am=1  11am=1 | 3  (Group3=2  Group4=1) | 0 | 5 | 5 | 2 | 3 |
| Day1 6pm | 23 | 10am=1  11am=1  noon=4  2pm=4  3pm=2  4pm=7  5pm=2 | 2  (Group3=1  Group4=1) | 2 | 6 | 3 | 3 | 7 |
| Day1 midnight | 53 | 11am=1  Noon=4  2pm=4  3pm=1  4pm=3  6pm=8  8pm=3  9pm=2  10pm=21  11pm=4 | 2  (Group4=1  Group3=1) | 10 | 9 | 8 | 10 | 14 |
| Day2 6am | 11 | Night day2=8  Day1 6pm=1  Day1 3pm=1 | 1  (Group4=1) | 0 | 3 | 2 | 0 | 5 |
| Day2 noon | 16 | 6am=1  8am=3  9am=1  10am=7  11am=2 | 2  (Group3=1  Group2=1) | 3 | 1 | 4 | 2 | 4 |
| Day2 6pm | 19 | 10am=1  11am=1  noon=3  1pm=1  2pm=8  4pm=3  5pm=1 | 1  (Group4=1) | 2 | 4 | 5 | 3 | 4 |
| Day2 midnight | 55 | 10am=1  noon=3  2pm=3  6pm=9  8pm=10  9pm=1  10pm=24  11pm=3 | 1  (Group4=1) | 13 | 8 | 10 | 13 | 10 |
| Day3 6am | 10 | night 3=8  day2 9pm=1 | 1  (Group4=1) | 0 | 5 | 0 | 0 | 4 |
| Day3 noon | 12 | 6am=1  8am=3  10am=3  11am=2 | 3  (Group4=1  Group2=2) | 1 | 1 | 2 | 2 | 3 |
| Day3 6pm | 21 | 11am=1  Noon=5  2pm=6  4pm=5  5pm=2 | 2  (Group4=1  Group2=1) | 1 | 4 | 3 | 3 | 8 |
| Day3 midnight | 53 | 11am=1  noon=3  2pm=2  4pm=3  5pm=1  6pm=8  8pm=9  10pm=25  11pm=1 | 1  (Group4=1) | 10 | 13 | 7 | 9 | 13 |
| Day4 6am | 13 | Day3 4pm=1  Day3 8pm=1  Night4=10 | 1  (Group4=1) | 1 | 3 | 0 | 1 | 7 |
| Day4 noon | 22 | 6am=4  7am=1  8am=5  10am=5  11am=1 | 6  (Group4=1  Group1=1  Group2=1  Group5=3) | 0 | 5 | 3 | 3 | 5 |
| Day4 6pm | 47 | 6am=3  8am=4  10am=2  noon=7  1pm=1  2pm=7  3pm=2  4pm=12  5pm=1  7pm=1  9pm=1 | 6  (Group4=1  Group1=1  Group2=1  Group5=3) | 3 | 10 | 7 | 10 | 11 |
| Day4 midnight | 80 | 6am=3  8am=3  10am=2  noon=8  2pm=7  3pm=1  4pm=11  5pm=1  6pm=11  7pm=1  8pm=4  9pm=1  10pm=21 | 6  (Group4=1  Group1=1  Group2=1  Group5=3) | 11 | 14 | 15 |  | 17 |

Further, for the secondary outcomes, missing data were not replaced, because it is assumed that the variables are missing at random. Further, only complete data sets were used for further analysis.

### Stopping Rules

We did not use a stopping rule and included patients until all groups had at least 24 participants.

### Significance

Two-sided p-values of *p* = .05 or less were considered to indicate statistical significance.

## Multiple Testing

To prevent accumulated α- errors, the analyses were Bonferroni corrected when necessary. Further, adequate post hoc tests were applied for all performed analyses (see 5.6).

# Summary of Study Data

The data were described with the help of tables and figures. The tables for summary data had the following structure: Columns for each treatment and overall order – APP, DOC, APP+DOC, TIME, and TAU annotated with the total population size relevant to that table/treatment, including any missing observations. All continuous variables will be summarised using the following descriptive statistics: n (non-missing sample size), mean, standard deviation. The frequency and percentages (based on the non-missing sample size) of observed levels was reported for all categorical measures. In general, all data were listed, sorted by site, treatment and subject, and when appropriate by visit number within subject. See 7.2. to retrace the analysis population for each figure and table.

## Subject Disposition

See Consort Flow Chart (see figure page 24)

During the assessment period, 787 patients received a TKR. First, the patients were screened on paper. If no obvious exclusion criteria were mentioned in the medical record in the available data, patients were screened in person and the study was explained to them. 120 patients were eligible for the study and decided to participate in the study. Hence, 120 patients were randomized to the five possible groups.

## Protocol Deviations

The summary statistics was performed as planned.

## Demographic and Baseline Variables

Demographic information and baseline variables were stated in section 5.6.

## Concurrent Illnesses and Medical Conditions

One exclusion criterion was mental illnesses according to ICD-10. Hence, patients with concurrent mental illnesses were excluded. The medical treatment (see 5.6) was adjusted according to medical conditions. Further, all patients were monitored closely after the surgery.

## Prior and Concurrent Medications

The postoperative medication consumption was protocolled and standardized (see section 5.6.). The medication prior to the study was assessed.

## Treatment Compliance

The treatment compliance was retraced by fulfilment of the questionnaires and the entries into the pain diary. Further, the consumption of morphine was monitored not only by the patients but also the nursing staff. In addition, the changes of the medication were retrieved from medical records.

# Efficacy Analyses

The summary table information was grouped as follows: Treatment groups, control groups. All analyses of the continuous efficacy variables (e.g., NRS pain score) was performed as analysis of variance with treatment group. Treatment groups were tested at the 2-sided 5% significance level. For the continuous variables, rANOVA (if applicable) was applied.

The primary population was the same as the analysis population (see section 7.2). To analyse the statistical difference, especially rANOVA was applied as the statistical procedure. Hence, the underlying statistical model for the analysis included measured of differences within and between subjects. The interaction was calculated between time of measurement and allocated group.

The null hypothesis for the primary outcome was that all means of the groups were equal. Hence, the null Hypothesis implied that the mean of the patients’ postoperative pain did not differ depending on the group membership. In contrast, the alternative hypothesis implied that at least two group means differed significantly from each other.

Hypothesis for all endpoints:

H_0_: μ_1_ = μ_2_ = μ_3_= μ_4_ = μ_5_ for μ = population mean

H_A_: At least two means are significantly different

The nature of our hypothesis is differences between groups. The applied methods to obtain parameter estimates are confidence intervals and p-values. The assumptions for rANOVA independent observations, normality and sphericity will be analysed accordingly. The assumption of independent observations was met because each case (row of data) in SPSS will be hold a different person. The normality assumption will be analysed with the help of a Q-Q Plots (if necessary). If the normality assumption will be violated, depending on the extent of deviation of normality, the data will be transformed accordingly. In case of equal big sample sizes (n_i_ > 10), ANOVA is robust against violations of assumptions (34). Last, the sphericity assumption will be analysed with the Mauchly’s test. If the sphericity assumption is violated, the degrees of freedom will be adjusted depending on the estimated sphericity epsilon (ε). The corrections will be based on the general guidelines. Hence, the Greenhouse-Geisser-correction will be applied if ε < 0.75 and the Huynh-Feldt correction will be applied if ε > 0.75.

Due to the within and between factors, rANOVA is the most appropriate statistical procedure. For the primary analysis missing data will be completed by using the LOCF. The full results are stored to enable future studies, meta-analysis, and replication of the analysis.

## Analyses of Assumptions

*Primary Outcome*

1. Pain: Course of pain, pain relief

Due to sample size (n_i_ = 24 and therefore > 10), normality did not have to be controlled for. However, Q-Q plots confirmed the normal distribution of the primary outcome. For the course of pain, the assumption of sphericity was violated, therefore the degrees of freedom will be Greenhouse-Geisser corrected (ε = 0.46). For pain relief, sphericity test was not necessary.

*Secondary Outcome*

1. Treatment Expectation

Due to sample size (n_i_ = 24 and therefore > 10), normality did not have to be controlled for. However, Q-Q plots confirmed the normal distribution. Due to only two time of measurements, the analysis for treatment expectation does not to be checked for sphericity.

1. Self-reported restriction on mobility

Due to sample size (n_i_ = 24 and therefore > 10), normality did not have to be controlled for. Due to only two time of measurements, the analysis for SETS did not have to be checked for sphericity.

1. Oxycodone consumption

Due to sample size (n_i_ = 24 and therefore > 10), normality did not have to be controlled for. For oxycodone consumption, the assumption of sphericity was violated, therefore the degrees of freedom were Greenhouse-Geisser corrected (ε = 0.61).

## Primary Efficacy Analysis

The primary efficacy analysis was to explore the course of postoperative pain for four days subsequent a TKR. The endpoints were postoperative pain at medication intake times (6am, 12noon, 6pm, and 12midnight) for four days starting the day after the surgery. Investigated were the group differences between the three treatment (APP, DOC, and APP+DOC) and two control groups (TAU and TIME). The summary statistics were produced in accordance with section 4.

## Secondary Efficacy Analyses

The secondary efficacy analyses investigated primarily the potential group differences of the primary efficacy analysis further by applying the post hoc tests. Further, the difference between post and preoperative pain were analysed with one-way ANOVA, the quantity of analgesics (oxycodone), subjective functional capacity, subjective treatment expectation and treatment success. The summary statistics were produced in accordance with section 5.2 and 4.

# Safety Analyses

The methods to describe the safety data in the final report were by tables and SPSS files and syntaxes.

## Extent of Exposure

Due to the reason that patients were stationary hospitalized for four days subsequent a TKR, patients received the according treatment for four days. After the treatment most of the patients were released from the clinic into rehabilitation centre to regain physical functions.

## Adverse Events

Adverse events were not anticipated due to the reason that the treatment was embedded into the usual care. Hence, the patients were closely monitored and in case of adverse events, the reasons and consequences were discussed. If the adverse event was a treatment emergent event, which was defined as an adverse event that occurred after the baseline assessment and worsen the post-treatment, it would have had to be decided if the planned treatment could have been continued. All adverse events which were related to the treatment were reported and included in the documentation.

In general, when calculating the incidence of adverse events, or any sub-classification thereof by treatment, time period, severity, etc., each subject was only be counted once and any repetitions of adverse events was ignored; the denominator was the total population size.

## Deaths, Serious Adverse Events and other Significant Adverse Events

Death, serious adverse events and other significant adverse events were monitored and treated in accordance with the situation.

## Pregnancies

Pregnancies were impossible, because the sample naturally was limited to post-menopausal subjects. Patients who received a TKR are 70 years old in average.

## Other Safety Measures

Due to the reason that the study took place in a stationary setting, the patients were monitored closely, and vital signs were measured regularly.

# Figures and Tables

*Figures*

To communicate the analyses results, figures were used. It was planned to include a figure of:

1. A flow chart according to CONSORT guidelines to portray the patient distribution.
2. The course of postoperative pain in a line diagram.
3. The course of the quantity of oxycodone in a line diagram.
4. The pain reduction in a bar graph.
5. The group features and design of study will be visualized.
6. A visual portray of the application.

*Tables*

To communicate the baseline data and medication plan, tables were used. It was planned to include tables of:

1. Medication plan according to national (German) guidelines
2. Baseline data

# Reporting Conventions

P-values ≥.001 to .01 were reported to 3 decimal places; p-values less than 0.001 were reported as “<0.001”; p-values >.01 were reported to 2 decimal places. The mean, standard deviation, and any other statistics other than quantiles, were reported to two decimal places, except in baseline characteristics in which they were portrait without decimal places.

# Technical Details

The analyses were made with SPSS 25.0 on a Windows Computer. The code was saved as a Syntax to be comprehensible in retrospect. The analyses were performed by one of the psychological researchers. Further, at the beginning of the syntaxes, there is a comment which includes the time, date, author, references to input, and output data.

# Listing of Tables, Listings and Figures

## Tables

| Table Title | Number | Population | Endpoint | Question-naire | Time points | Covariate or  Subgroups | Summary Statistic | Formal  Analysis |
| --- | --- | --- | --- | --- | --- | --- | --- | --- |
| Demographics | 1.1 | All | Gender |  | Baseline | Treatment | Mean (SD) | N.A. |
|  |  |  | Female sex |  | Baseline | Treatment | No. (%) | N.A. |
| Baseline | 1.2 | All | **Knee Pain** |  |  |  |  |  |
|  |  |  | Clinical Pain without analgesics |  | Baseline | Treatment | Mean (SD) | N.A. |
|  |  |  | Clinical Pain with analgesics |  | Baseline | Treatment | Mean (SD) | N.A. |
|  |  |  | Acute Pain |  | Baseline | Treatment | Mean (SD) | N.A. |
|  |  |  | Acute Pain |  | Baseline | Treatment | Mean (SD) | N.A. |
|  |  |  | **Expectations** |  |  |  |  |  |
|  |  |  | Expected postoperative knee pain  with analgesics |  | Baseline | Treatment | Mean (SD) | N.A. |
|  |  |  | Expected postoperative knee pain  without analgesics |  | Baseline | Treatment | Mean (SD) | N.A. |
|  |  |  | Expected postoperative wound pain  with analgesics |  | Baseline | Treatment | Mean (SD) | N.A. |
|  |  |  | Expected postoperative wound pain  without analgesics |  | Baseline | Treatment | Mean (SD) | N.A. |
|  |  |  | Expected pain in 6-9 months without analgesics |  | Baseline | Treatment | Mean (SD) | N.A. |
|  |  |  | Expected pain in 6-9 months with analgesics |  | Baseline | Treatment | Mean (SD) | N.A. |
|  |  |  | Restriction in mobility with analgesics |  | Baseline | Treatment | Mean (SD)) | N.A. |
|  |  |  | ***Subjective functional capacity*** |  |  |  |  |  |
|  |  |  | Subjective functional capacity without analgesics |  | Baseline | Treatment | Mean (SD) | N.A. |
|  |  |  | Subjective functional capacity with analgesics |  | Baseline | Treatment | Mean (SD) | N.A. |
|  |  |  | ***Expectations*** |  |  |  |  |  |
|  |  |  | Expected subjective functional capacity without analgesics |  | Baseline | Treatment | Mean (SD) | N.A. |
|  |  |  | Expected subjective functional capacity with analgesics |  | Baseline | Treatment | Mean (SD) | N.A. |
|  |  |  | Expected pain reduction through analgesics |  | Baseline | Treatment | Mean (SD) | N.A. |
|  |  |  | Lequesne (Mobility) | Lequesne | Baseline | Treatment | Mean (SD) | N.A. |
|  |  |  | PHQ-4 Questionnaire |  |  |  |  |  |
|  |  |  | PHQ-2 | PHQ-4 | Baseline | Treatment | Mean (SD) | N.A. |
|  |  |  | GAD-2 | PHQ-4 | Baseline | Treatment | Mean (SD)) | N.A. |
|  |  |  | FSS Questionnaire |  |  |  |  |  |
|  |  |  | Catastrophizing | FSS | Baseline | Treatment | Mean (SD) | N.A. |
|  |  |  | Act Coping | FSS | Baseline | Treatment | Mean (SD)) | N.A |
|  |  |  | Stanford Expectation of Treatment Scale (SETS) |  |  |  |  |  |
|  |  |  | Positive expected treatment effectiveness (analgesics) | SETS | Baseline | Treatment | Mean (SD) | N.A. |
|  |  |  | Negative expected treatment effectiveness (analgesics) | SETS | Baseline | Treatment | Mean (SD) | N.A. |
| Course of  postoperative pain | 2.1 | All | Postoperative pain for four days subsequent TKR | Pain diary | Four days  subsequent TKR | Treatment | n, mean, min, max | rANOVA |
| Quantity of  oxycodone conusmption | 2.2 | All | Oxycodone consumption | Patient record/  Pain diary | Four days  subsequent TKR | Treatment | n, mean, min, max | rANOVA |
| Subjective  functional capacity | 2.3 | All | Functional capacity |  | Four days after TKR | Treatment | n, mean, min, max | rANOVA |
| Treatment success | 2.5 | All | Positive subjective treatment success (analgesics) | SETS | Four days after TKR | Treatment | n, mean, min, max | rANOVA |
|  |  |  | Negative subjective treatment success (analgesics) | SETS | Four days after TKR | Treatment | n, mean, min, max | rANOVA |
|  | | | | | | | | |

## Figures

1. The first figure will be a flow chart according to CONSORT guidelines to portray the patient distribution (see 8.1).


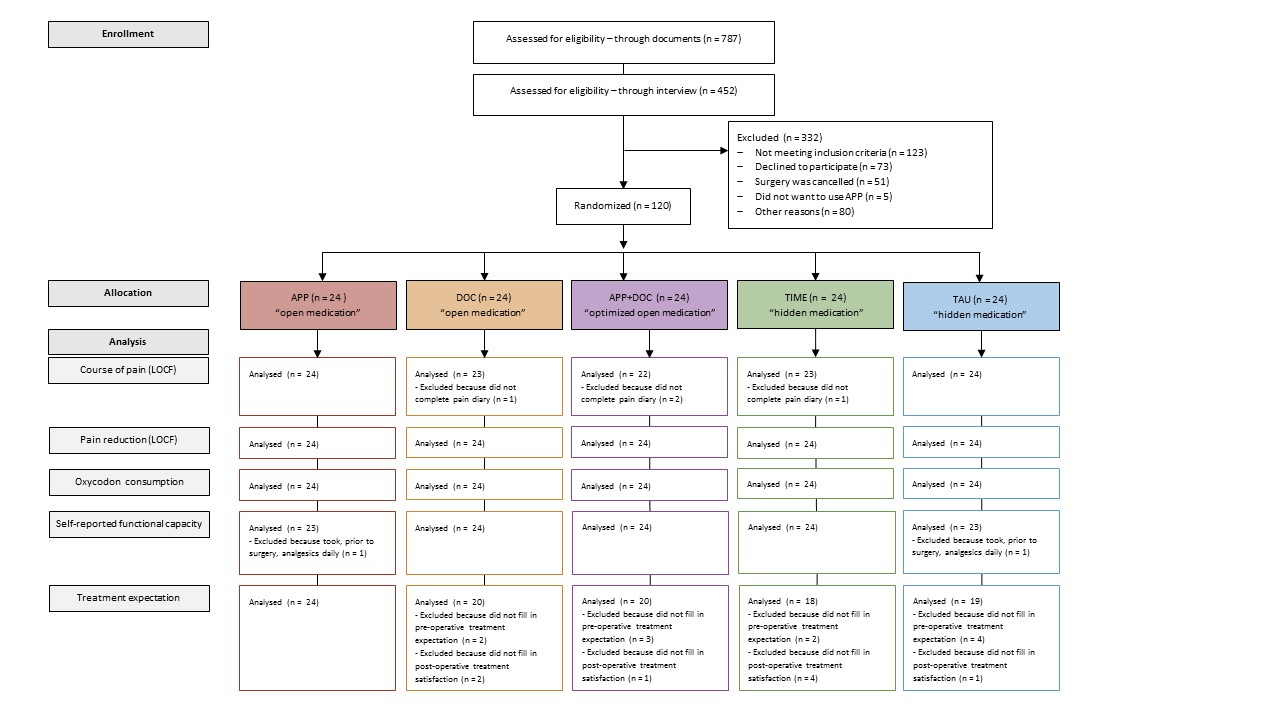


1. The course of postoperative pain in a line diagram (Example Figure).

| **Subject** | **Description** |
| --- | --- |
| **Title** | Course of postoperative pain |
| **Footnotes** | Figure x. Course of postoperative pain for four days subsequent TKR. The reference times are the medication intake times (6am, noon, 6pm, and midnight). |
| **Numbering** | Figure 1 |
| **Population** | Analysis population (see 7.4) |
| **Type of figure** | Line graph |
| **Co-ordinates** | Horizontal co-ordinates = NRS 0-10  Vertical co-ordinates = Time references (6am, noon, 6pm, midnight) |
| **Statistic(s)** | rANOVA |
| **Sub plots** | Univariate ANOVA for daily analysis |

1. The course of the quantity of oxycodone (Example Figure).

| **Subject** | **Description** |
| --- | --- |
| **Title** | Oxycodone consumption |
| **Footnotes** | Figure x. Oxycodone consumption for four days subsequent the TKR. |
| **Numbering** | Figure 2 |
| **Population** | Analysis population (see 7.4) |
| **Type of figure** | Line graph |
| **Co-ordinates** | Horizontal co-ordinates = Oxycodone consumption in mg  Vertical co-ordinates = Days |
| **Statistic(s)** | rANOVA |
| **Sub plots** | Univariate ANOVA for daily analysis |

1. A visual portray of the application


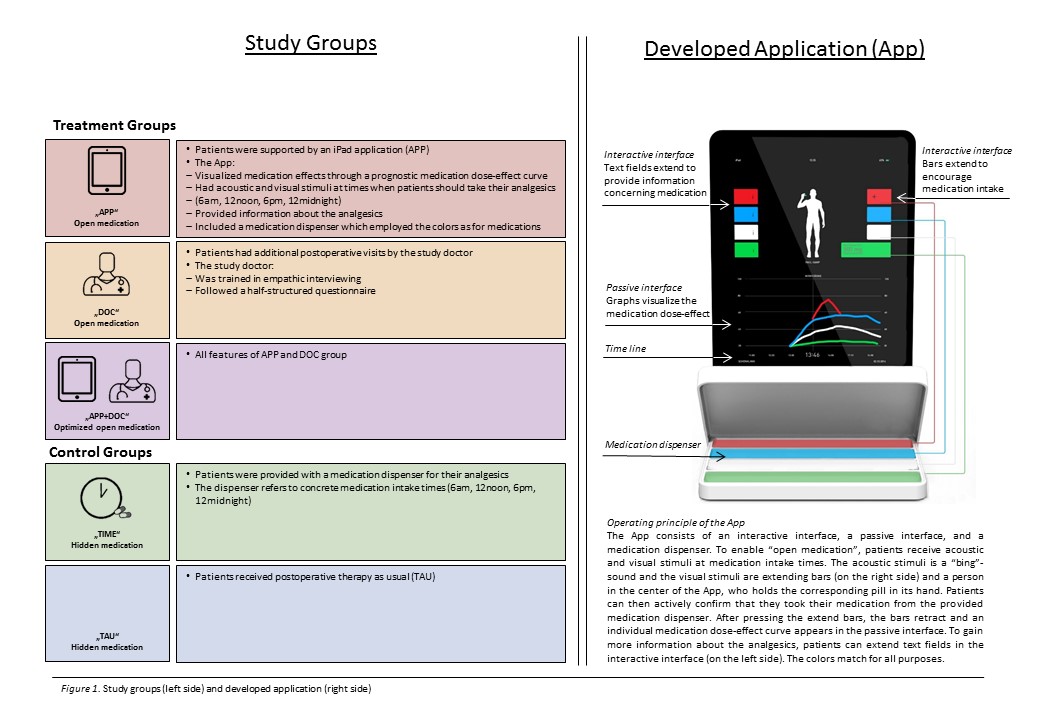


## Listing Tables and Figures

The following is a listing of figures.

| **Title** | **Number** | **Population** | **Figure/ Table** |
| --- | --- | --- | --- |
| Medication plan | 1 | N.A. | Table |
| Demographics and Baseline | 2 | Full Analysis | Table |
| Application | 3.1 | N.A. | Figure |
| Group overview | 3.2 | N.A. | Figure |
| CONSORT | 4 | Full Analysis | Figure |
| Course of Postoperative pain | 5.1 | Full Analysis | Figure/ Table |
| Oxycodone Consumption | 5.2 | Full Analysis | Figure/ Table |
