# Supplementary figures and images for: A digital application and augmented physician rounds reduce postoperative pain and opioid consumption after primary total knee replacement (TKR): a randomized clinical trial
Source: BMC Med. 2022 Dec 5;20:469. doi: 10.1186/s12916-022-02638-0 (PMC9721029; doi:10.1186/s12916-022-02638-0)

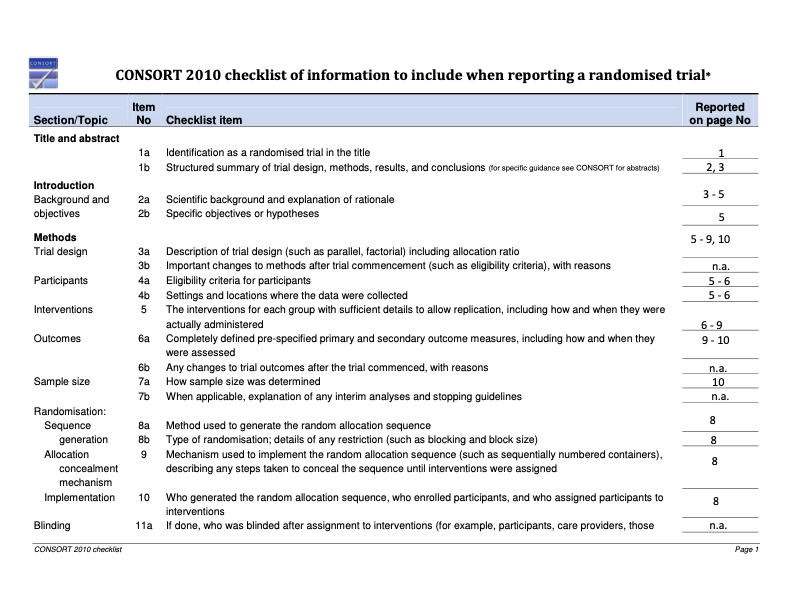

Supplement: Supplementary file 4 — Additional file 4: Doc S4. CONSORT Checklist. [file 12916_2022_2638_MOESM4_ESM.tiff]
